# Isolation and Comparative Genomic Analysis of Reuterin-Producing *Lactobacillus reuteri* From the Chicken Gastrointestinal Tract

**DOI:** 10.3389/fmicb.2020.01166

**Published:** 2020-06-04

**Authors:** Anna Greppi, Paul T. Asare, Clarissa Schwab, Niklaus Zemp, Roger Stephan, Christophe Lacroix

**Affiliations:** ^1^Institute of Food, Nutrition and Health, Laboratory of Food Biotechnology, Department of Health Sciences and Technology, ETH Zurich, Zurich, Switzerland; ^2^Genetic Diversity Centre, Department of Environmental Systems Sciences, ETH Zurich, Zurich, Switzerland; ^3^Institute for Food Safety and Hygiene, University of Zurich, Zurich, Switzerland

**Keywords:** reuterin, antimicrobial, chicken, *Lactobacillus reuteri*, comparative genomics

## Abstract

*Lactobacillus reuteri* is a natural inhabitant of selected animal and human gastrointestinal tract (GIT). Certain strains have the capacity to transform glycerol to 3-hydroxypropionaldehyde (3-HPA), further excreted to form reuterin, a potent antimicrobial system. Reuterin-producing strains may be applied as a natural antimicrobial in feed to prevent pathogen colonization of animals, such as in chicken, and replace added antimicrobials. To date, only seven *L. reuteri* strains isolated from chicken have been characterized which limits phylogenetic studies and host-microbes interactions characterization. This study aimed to isolate *L. reuteri* strains from chicken GIT and to characterize their reuterin production and antimicrobial resistance (AMR) profiles using phenotypic and genetic methods. Seventy strains were isolated from faces, crops and ceca of six chicken from poultry farms and samples from slaughterhouse. Twenty-five strains were selected for further characterization. Draft genomes were generated for the new 25 isolates and integrated into a phylogenetic tree of 40 strains from different hosts. Phylogenetic analysis based on gene content as well as on core genomes showed grouping of the selected 25 *L. reuteri* chicken isolates within the poultry/human lineage VI. Strains harboring *pdu-cob-cbi-hem* genes (23/25) produced between 156 mM ± 11 and 330 mM ± 14 3-HPA, from 600 mM of glycerol, in the conditions of the test. All 25 chicken strains were sensitive to cefotaxime (MIC between 0.016 and 1 μg/mL) and penicillin (MIC between 0.02 and 4 μg/mL). Akin to the reference strains DSM20016 and SD2112, the novel isolates were resistant to penicillin, possibly associated with identified point mutations in *ponA*, *pbpX*, *pbpF* and *pbpB*. All strains resistant to erythromycin (4/27) carried the *ermB* gene, and it was only present in chicken strains. All strains resistant to tetracycline (5/27) harbored *tetW* gene. This study confirms the evolutionary history of poultry/human lineage VI and identifies *pdu-cob-cbi-hem* as a frequent trait but not always present in this lineage. *L. reuteri* chicken strains producing high 3-HPA yield may have potential to prevent enteropathogen colonization of chicken.

## Introduction

*Lactobacillus reuteri* inhabits the gastrointestinal tract (GIT) of selected animals where it forms biofilms on the non-glandular, squamous epithelium lining the upper GIT. In poultry, *L. reuteri* is the most abundant *Lactobacillus* species in the GIT, mainly found in the crop and the cecum (Wang et al., 2014). Distinct phylogenetic lineages of *L. reuteri* are coherent with host origin, reflecting co-evolution of this species with the vertebrate hosts (Oh et al., 2010). The evolutionary adaptation differentiates the species in host-adapted phylogenetic lineages comprised of isolates from rodents (lineages I and III), humans (lineage II), pigs (lineages IV and V) and poultry/human (lineage VI) (Oh et al., 2010; Spinler et al., 2014). Newly identified herbivore strains isolated from goat, sheep, cow, and horse have not yet been assigned to phylogenetic lineages (Yu et al., 2018). Host adaption has been linked to the occurrence of specific functional traits, e.g., rodent *L. reuteri* isolates possess the genes responsible for the synthesis of urease, as the strains are exposed continuously to urea in the forestomach of mice (Walter et al., 2011). In case of herbivore isolates, specific genes involved in porphyrin and chlorophyll metabolism and biosynthesis of amino acids have been identified, indicating possible host-adaptation mechanisms involving amino acids biosynthesis in herbivores (Yu et al., 2018).

Genomes of poultry and human *L. reuteri* isolates (lineages II and VI) have been shown to harbor the *pdu-cbi-cob-hem* operon, as a lineage-specific trait (Frese et al., 2011). This operon contains genes for glycerol and propanediol utilization (*pdu*) and cobalamin biosynthesis (*cbi-cob*), *hem* genes and some accessory genes. Cobalamin is a co-factor for glycerol/diol dehydratase PduCDE (EC 4.2.1.30). PduCDE catalyses the conversion of 1,2-propanediol to propanal, which can be further metabolized by other enzymes of the *pdu* operon to propanol or propionate (Engels et al., 2016a). Glycerol, a second substrate of PduCDE, is transformed into the intermediate 3-hydroxypropionaldehyde (3-HPA) which can be further metabolized to 1,3-propanediol or 3-hydroxypropionate (Gänzle, 2015). 3-HPA produced from glycerol is released from the cell forming the dynamic multi-compound reuterin system, with broad antimicrobial spectrum and consisting of 3-HPA, its hydrate and dimer and acrolein (Stevens et al., 2010; Engels et al., 2016b). Acrolein, a highly reactive toxicant, was recently shown to be the main component for the antimicrobial activity of reuterin (Engels et al., 2016b; Asare et al., 2018).

Due to the high persistence of *L. reuteri* in the chicken GIT and the established antimicrobial activity of reuterin, *L. reuteri* has high potential to be applied as a natural antimicrobial in feed to prevent pathogen infection of chicken (Hou et al., 2015). *L. reuteri* strains isolated from chicken intestine were shown effective against *Salmonella* spp. and *Escherichia coli* resistant to various antibiotics (Nitisinprasert et al., 2000). Moreover, *L. reuteri* in the early post-hatching period appear to modify the composition of ileum microbiota of broiler chicken, resulting in enrichment of potentially beneficial lactobacilli and suppression of Proteobacteria (Nakphaichit et al., 2011). To select functional *L. reuteri* strains, a key trait is the determination of their antimicrobial resistance (AMR) profiles to identify intrinsic and extrinsic resistances that may be potentially transferred. Lactobacilli are known to be intrinsically resistant against vancomycin. However, the occurrence of tetracycline and erythromycin genes on mobile elements has been reported for different *Lactobacillus* spp. (Egervärn et al., 2009).

As of April 2018, among the complete genomes of *L. reuteri* deposited in the National Center for Biotechnology Information (NCBI), only seven strains were isolated from chicken, representing only 4% of all available NCBI *L. reuteri* genomes (*n* = 187) and thus limiting any phylogenetic analysis and host-microbes adaptation studies. The majority of the NCBI *L. reuteri* deposited genomes comes from strains which had been isolated from mouse (47), human (19) and pig (28), while few originated from sourdough (7), chicken (7), goat (5), cow (5), rat (4) sheep (4), dairy and fermented products (4), horse (3), probiotic capsule (1), and wine (1).

It was, therefore, the aim of this study to isolate and characterize *L. reuteri* strains from chicken and characterize their reuterin production and AMR profiles using phenotypic and genotypic methods. Draft genomes of the isolates were analyzed combined with 40 *L. reuteri* genomes of strains previously isolated from different hosts to assess genetic diversity and gain insight into distinguishing features related to chicken and enrich previously phylogenetic characterization of *L. reuteri*.

## Materials and Methods

### Bacterial Strains and Growth Conditions

*L. reuteri* DSM20016 (Leibniz Institute DSMZ-German Collection of Microorganisms and Cell Cultures, Braunschweig, Germany) and *L. reuteri* SD2112 (BioGaia AB, Stockholm, Sweden) were used as reference strains. Reference strains, as well as all *L. reuteri* isolated in this study, were propagated anaerobically (Oxoid, AnaeroGen^TM^, Basingstoke, United Kingdom) at 37°C in de Man, Rogosa and Sharpe (MRS) broth medium (Biolife, Milan, Italy).

### Bacterial Isolation

Six Lohmann brown layer chicken (13 weeks old) were obtained from six chicken farms in Switzerland. Chicken samples were taken under the supervision of a veterinarian, as per Swiss animal handling regulation. Ceca and feces were aseptically collected. In parallel, 10 whole gut of Cobb 500 broiler chicken were obtained from Schönholzer Werner abattoir in Wädenswil (Zurich) and transported to the lab within 1 h.

*L. reuteri* strains were isolated from the chicken GIT samples using the protocol previously described (Walter et al., 2011), with some modifications. Briefly, 1 g of crop, cecal or fecal content was added to 10 mL of sterile phosphate buffer saline (PBS) (137 mM NaCl, 2.7 mM KCl, 10 mM Na_2_HPO_4_, 2 mM KH_2_PO_4_, pH 7.4) and homogenized in a stomacher (BagMixer^®^ 400 P, Interscience, Saint Nom, France) at high speed for 1 min. To ensure the initial cultivation of *L. reuteri* from chicken crop and feces, the modified MRS medium (mMRS) was used, containing maltose, the preferred substrate of heterofermentative lactobacilli, and fructose as electron acceptor (Zhao and Gänzle, 2018). The mMRS contained (g/l): tryptone, 10; yeast extract, 5; meat extract, 5; maltose, 10; fructose, 5; K_2_HPO_4_.3H_2_0, 2.6; KH_2_PO_4_, 4; cysteine-HC1, 0.5; NH4CI, 3; Tween 80, 1 (Stolz et al., 1995; Zhao and Gänzle, 2018). Suspensions from samples were serially diluted and spread on mMRS. The agar plates were incubated overnight at 42°C under anaerobic condition using AnaeroGen 2.5 L (Thermo Fisher Diagnostics AG, Pratteln, Switzerland). Replica plates were prepared using Scienceware replica plater and velveteen squares (Sigma-Aldrich, Buchs, Switzerland), and incubated overnight as presented above. After incubation, one plate was overlaid with 500 mM glycerol agar (1% agar) and incubated at 37°C for 30 min for testing reuterin production of colonies. A colourimetric method was used with the addition of 5 mL 2,4-dinitrophenylhydrazine (0.1% in 2 M HCl), 3 min incubation, removal of the solution, and addition of 5 mL 5M KOH. *L. reuteri* colonies showing purple zones indicating reuterin synthesis were streaked on MRS agar plates, and single colony were subcultured three times in MRS broth (1% inoculum, 18 h at 42°C). Few colonies which show a colony morphology of *L. reuteri* but no purple zone were picked as negative controls. Species confirmation and reuterin production quantification was performed for both positive and selected negative selected colonies. The strains were named PTA X, with X indicating the chicken ID, with no number being assigned to the abattoir sample; F and C indicate isolating from feces and cecum, respectively, followed by the number of the isolated strains.

### Bacterial Identification

Genomic DNA was isolated using a lysozyme-based cell wall digestion followed by the Wizard genomic DNA purification kit (Promega, Dübendorf, Switzerland). Total DNA was quantified by absorbance at 260 nm using NanoDrop^®^ ND-1000 Spectrophotometer (Witec AG, Littau, Switzerland). The DNA quality was analyzed by electrophoresis in 1.2% (w/v) agarose gel and Gel Red staining (VWR International AG, Dietikon, Switzerland). The DNA samples were stored at −20°C until further analysis.

To confirm the identity of the isolates, the 1.6 kbp full region of 16S rRNA gene was amplified by PCR using universal primers bak4 (5′-AGGAGGTGATCCARCCGCA-3′) and bak11w (5′-AGTTTGATCMTGGCTCAG -3′) (Greisen et al., 1994; Goldenberger et al., 1995). The 16S rRNA PCR assay consisted of 5 min at 95°C, followed by 35 cycles of 15 s at 95°C, 30 s at 60°C and 2 min at 72°C and final extension for 7 min at 72°C. Sanger sequencing of the PCR amplicon was performed at GATC (Konstanz, Germany). To identify the closest homologs, DNA sequences obtained were aligned using the Basic Local Alignment Search Tool (BLAST) (Altschul et al., 1990). Sequence with 100% homology was used to identify *L. reuteri*.

### Strain Typing

The enterobacterial repetitive intergenic consensus (ERIC) sequence was used to differentiate between isolates, as ERIC has the ability to discriminate *Lactobacillus reuteri* isolates to species and strain levels (Stephenson et al., 2009). The ERIC-PCR assay was performed using ERIC1R (5′-ATGTAAGCTCCTGGGGATTCAC-3′) and ERIC2 (5′-AAGTAAGTGACTGGGGTGAGCG-3′) primers (Versalovic et al., 1991; Ventura and Zink, 2002; Stephenson et al., 2010). The ERIC-PCR assay was performed using 100 ng of template DNA of each isolate. The protocol consisted of 7 min at 95°C, followed by 30 cycles of 30 s at 90°C, 1 min at 52°C, and 8 min at 65°C, and a final extension for 16 min at 65°C. The ERIC-PCR amplicons were analyzed on 2% (wt/vol) agarose gels for 6 h at 60 Volts. GeneRuler DNA ladder mix (Fermentas, Le Mont-sur-Lausanne, Switzerland) was used as a molecular size marker according to the manufacturer’s directions, and gels were visualized by Gel Red staining. Gels were analyzed using Gel Compar II version 6.5 software package (Applied Maths, Sint-Martens-Latem, Belgium). *L. reuteri* strains with unique ERIC profiles were visually selected, and amplicons re-run on a single agarose gel. A dendrogram of similarity was generated from the gel with selected isolates using the Pearson correlation similarity coefficient and the unweighted-pair group method (UPGMA) with arithmetic averages, and 1% optimization. A cutoff of 84% similarity was used to define the “ERIC type.” Based on the clustering obtained, confirmed isolates with unique ERIC type were selected for whole-genome sequencing and characterization.

### Generation and Annotation of Draft Genomes

Genomic DNA from the isolated strains was obtained as described above and standardized to 100 ng/μL. The whole genomes of 25 *L. reuteri* isolates were sequenced with the standard set of 96 Illumina paired-end barcodes on a HiSeq 2500 Illumina Technology (Illumina Inc., San Diego, CA, United States) with 2× 125 high output mode. The genomic library was generated using reagents from NEBNext Illumina preparation kit. Raw paired-end reads were quality trimmed using the default settings of Trimmomatic (Bolger et al., 2014). Read pairs were merged by FLASH (Mag and Salzberg, 2011). *De novo* assembly was performed with SPAdes assembler (version 3.12) with *–careful* option (Bankevich et al., 2012). The quality of the assembly based on evolutionarily informed expectations of gene content from near-universal single-copy orthologs selected from OrthoDB v9 was assessed by BUSCO (Simão et al., 2015) and QUAST (Gurevich et al., 2013). Reference guided ordering of scaffolds based on iterative alignment steps was performed by QUAST using *L. reuteri* DSM20016 genome as a reference. SeqKit (Shen et al., 2016) and QUAST were used to retrieve genome features. The complete genome of *L. reuteri* DSM20116 type strain was compared individually with each of the 25 draft genomes of *L. reuteri* chicken isolates. Average nucleotide identity (ANI) values between two genomic datasets were calculated using JSpecies (Richter et al., 2016). Genomes with ANI values above 95% were considered as belonging to the same species (Goris et al., 2007).

Twenty-five *L. reuteri* draft genomes (this study) and 40 NCBI deposited *L. reuteri* genomes of strains isolated from different hosts (chicken, human, mouse, rat, pig, sourdough, goat, sheep, cow, horse; Supplementary Table S1) were structurally annotated using the PROKKA 3.10.1 suite (Seemann, 2014). The 40 NCBI complete genomes were chosen to cover host diversity of *L. reuteri*, and included genomes previously used in comparative studies (Oh et al., 2010; Duar et al., 2017) and all available genomes from chicken isolates at the time of data analysis (April 2018). Only contigs higher than 500 bp were included in the analysis, and *Lactobacillus* was selected as the reference database for the annotation (options used: *–genus* Lactobacillus *–species* reuteri *–usegenus* Lactobacillus *–mincontiglen* 500).

### Comparative Genomics

Comparative genome analysis was based on gene content tree, core genome phylogenetic tree and a nucleotide-content similarity matrix (ANI matrix). For the generation of the gene content tree, a matrix based on gene content (binary data for presence or absence of each annotated gene) was generated comprising all annotated genes of 25 *L. reuteri* draft genomes plus the 40 *L. reuteri* NCBI genomes. The gene content tree was then constructed using the hierarchical cluster analysis (hcust) on R 3.4.4 while a core genome phylogenetic tree was calculated using EDGAR 2.3 (Blom et al., 2016). Concatenated sequences were used to calculate a distance matrix which provided the input for the neighbor-joining method with PHYLIP implementation. EDGAR 2.3 calculated the core genome of each identified clusters as a set of orthologous genes present in all strains belonging to each cluster. For the generation of the similarity matrix, ANI was calculated on EDGAR 2.3 with all-against-all comparisons at the nucleotide level for all 65 *L. reuteri* strains.

Identified lineages were named based on host origin of the strains, and lineages specific features were determined using “Define metacontigs” function in EDGAR 2.3. Core groups were created for the sets of strains derived from the phylogenetic analysis. Venn diagrams were designed in EDGAR to identify and count the number of shared genes of the core genomes of identified clusters of strains (cluster A, B and C) as well as between human VI and poultry VI strains, human VI and human II strains and poultry lineage VI, human lineage VI and human lineage II of *L. reuteri* strains. Unique genes of poultry/human lineage VI were manually categorized based on UniProt protein description into the following groups: transport proteins, DNA-binding proteins, transferases, lyases, oxidoreductases, membrane proteins, hydrolases, virulence-related proteins, RNA-binding and prophage-related proteins. The presence of genes of the *pdu-cob-hem-cbi* cluster was manually checked, and a heatmap of presence/absence of genes of interest was designed with GraphPad Prism 8.0 (GraphPad Software Inc. La Jolla California, United States). For each of the reuterin-positive *L. reuteri* strains, the nucleotide sequence of *pduA*, *pduB*, *pduC*, *pduD* and *pduE* was extracted and concatenated. Concatenated sequences were used to construct a phylogenetic tree with the Neighbor-Joining Method and Tamura-Nei genetic distance model using MEGA version X (Kumar et al., 2018). The resulting trees were rooted using pduABCDE genes of *Salmonella Typhimurium* SL1344 as outgroup. By using the same method, individual phylogenetic trees per each of the pdu genes were also built.

The presence of AMR genes in the assembled genomes was also manually checked and integrated with the results from ResFinder 3.0 tool (Zankari et al., 2012). A database was created with all (*n* = 17) NCBI deposited *L. reuteri* plasmid sequences. The database was used to determine location of extracted *ermB* and *tetW* gene sequences associated with resistance phenotypes, by NCBI Blastn alignment. AMR genes that aligned completely with more than 99% identity with one of the plasmids of the database, were considered plasmid located.

For penicillin-resistant *L. reuteri* strains, point mutations (SNPs) in penicillin-binding proteins genes *ponA*, *pbpX*, *pbpF* and *pbpB* were checked by aligning deduced amino acid sequences of resistant and sensitive strains with NCBI-deposited sequences for *Lactobacillus* in Geneious 9.1.8.

### PCR for *tetW* and *ermB* Detection

PCR for *tetW* and *ermB* genes was performed to confirm the genomic data. *TetW* was amplified with tetw-rev and tetw-fw primers (Kastner et al., 2006; Egervärn et al., 2009). The PCR protocol comprised 35 cycles of 95°C for 30 s, 56°C 45 s and 72°C 30 s. The *ermB* gene was amplified with primers ermA1 (5′-TCTAAAAAGCATGTAAAAGAA-3′) and ermA2 (5′-CTTCGATAGTTTATTAATATTAGT-3′) using 30 PCR cycles composed of 95°C for 30 s, 52°C 45 s, 72°C 2 min (Egervärn et al., 2009).

### Reuterin Production

Reuterin production was determined using a two-step process (Stevens et al., 2013). Cell pellets obtained from 16 h *L. reuteri* cultures (OD_600_ ≈ 8.0) were collected by centrifugation, resuspended in 600 mM glycerol solution and incubated at 25°C for 2 h. For reuterin biosynthesis, the concentrations of glycerol, 3-HPA and acrolein were measured by High Performance Liquid Chromatography using Refractive Index (HPLC-IR, Hitachi LaChrome, Merck, Dietikon, Switzerland) and Ion-Exclusion Chromatography with Pulsed-Amperometric Detection (IC-PAD) analysis, respectively (Engels et al., 2016b; Asare et al., 2018). For HPLC-RI, an Aminex HPx-87H column and sulfuric acid (10 mM) was used as eluent. The IC-PAD Thermo Scientific (Reinach, Switzerland) ICS-5000+ system was equipped with a quaternary gradient pump, a thermostated autosampler and an electrochemical detector with a cell containing an Ag/AgCl reference electrode and a disposable thin-film platinum working electrode tempered at 25°C. Analytes were separated with a Thermo Scientific IonPac ICE-AS1 4× 250 mm ion-exclusion column with a guard column, operated at 30°C. The solvent system was isocratic 0.1 M methanesulfonic acid at 0.2 mL/min for 36 min.

### Antimicrobial Susceptibility Profiling

*L. reuteri* chicken isolates and reference strains *L. reuteri* SD2112 and DSM20016 were tested for susceptibility to cefotaxime (CFX), erythromycin (ERM), penicillin (PEN), tetracycline (TET), vancomycin (VAN), and ciprofloxacin (CIP), using gradient diffusion MTS^TM^ strips (Liofilchem, Roseto degli Abruzzi, Italy). These antibiotic were selected as they belong to World Organisation for Animal Health (OIE) list of antimicrobials of veterinary importance, and to the World Health Organisation (WHO) list of antimicrobials of critical importance for human health (World Organisation for Animal Health [OIE], 2015; World Health Organisation [WHO], 2019). In particular, one antibiotic per critically important antibiotic family was tested: (i) quinolone family (CIP), (ii) tetracycline family (TET), (iii) glycopeptides family (VAN); (iv) B-lactam family (PEN); (v) macrolide family (ERM); (vi) cephalosporin family (CFX). The concentration range tested was 0.016 to 256 μg/mg, except for CIP (0.002 to 32 μg/mL). Briefly, 16 h overnight *L. reuteri* cultures were added to a sterile MRS broth (1% v/v) and incubated at 37°C for 6 h. The bacterial cultures were standardized to OD_600_ 1.0 (corresponding to approximately 10^8^ CFU/mL) using a PowerWave XS microplate spectrophotometer (BioTek, Sursee, Switzerland). The diluted culture (10^6^ CFU/mL) was evenly swabbed on MRS agar plates in duplicate using a sterile cotton bud. MTS^TM^ strips were placed on the surface of the agar and incubated at 37°C for 24 h in anaerobic condition supplied by the gas package (AnaeroGen, Thermo Fisher Diagnostics AG, Pratteln, Switzerland). The minimum inhibitory concentration (MIC) was recorded as the point where inhibition curves intersect the scale on the MTS^TM^ strip.

### Data Accession Number

The draft genomes of 25 *L. reuteri* strains have been deposited in NCBI under the accession numbers indicated in Table 1. This Whole Genome Shotgun project has been deposited at DDBJ/ENA/GenBank under the accession PRJNA473635.

**TABLE 1:** Draft genome features of 25 *L. reuteri* strains isolated from chicken GIT and sequenced in this study.

Strain	Animal	Sampling spot	Genome size (Mbp)	Contig no. ^1^	N50	GC content (%)	No. of CDS	ANI^2^	tmRNA	tRNA	rRNA	NCBI accession
PTA5_11	A	Crop	2.13	151	27,612	38.48	2081	95.83	1	53	3	QKQO00000000
PTA8_1	A	Crop	2.15	149	26,824	38.47	2089	95.81	1	45	3	QKQN00000000
PTA1_C1	B	Cecum	2.05	140	32,611	38.71	1980	95.20	1	64	3	QKQM00000000
PTA1_C3	B	Cecum	2.02	127	33,165	38.8	1974	95.08	1	65	3	QKQL00000000
PTA1_C4	B	Cecum	2.02	97	50,527	38.8	1957	95.01	1	52	3	QKQK00000000
PTA1_F3	B	Feces	2.06	108	45,078	38.79	2000	95.33	1	64	3	QKQJ00000000
PTA2_C2	C	Cecum	2.15	112	40,818	38.66	2129	95.63	1	59	6	QKQI00000000
PTA4_C1	D	Cecum	2.05	103	35,549	38.83	1996	95.19	1	58	3	QKQH00000000
PTA4_C2	D	Cecum	2.06	116	33,725	38.71	2021	95.12	1	64	3	QKQG00000000
PTA4_C4	D	Cecum	2.07	68	69,730	38.55	2050	95.13	1	61	3	QKQF00000000
PTA5_F1	E	Feces	2.04	125	39,198	38.78	1954	95.20	1	64	3	QKQE00000000
PTA5_F4	E	Feces	2.11	146	32,910	38.69	2052	95.27	1	61	3	QKQD00000000
PTA5_F11	E	Feces	1.96	126	32,904	38.76	1909	95.15	1	63	3	QKQC00000000
PTA5_F13	E	Feces	2.13	108	50,846	38.59	2144	95.07	1	60	3	QKQB00000000
PTA5_C4	E	Cecum	2.06	140	32,458	38.69	1995	95.04	1	64	3	QKQA00000000
PTA5_C5	E	Cecum	2.07	105	59,278	38.76	2007	95.27	1	58	3	QKPZ00000000
PTA5_C6B	E	Cecum	2.71	657	22,978	38.7	2298	95.26	1	68	4	QKPY00000000
PTA5_C13	E	Cecum	2.19	158	34,750	38.84	2002	95.16	1	64	3	QKPX00000000
PTA6_C2	F	Cecum	2.23	191	34,786	38.86	2009	95.27	1	63	3	QKPW00000000
PTA6_C7	F	Cecum	2.19	154	39,778	38.74	2038	95.25	1	52	4	QKPV00000000
PTA6_F1	F	Feces	2.28	219	31,238	38.88	2003	95.20	1	64	3	QKPU00000000
PTA6_F2	F	Feces	2.17	147	45,484	38.59	2032	95.15	1	65	3	QKPT00000000
PTA6_F4	F	Feces	2.57	442	40,200	38.57	2194	95.18	1	67	5	QKPS00000000
PTA6_F6	F	Feces	2.47	414	26,145	38.74	2126	95.23	1	66	6	QKPR00000000
PTA6_F8	F	Feces	2.04	135	39,822	38.76	1983	95.30	1	60	3	QKPQ00000000

*^1^ >500 bp. ^2^Average Nucleotide Identity based on BLAST+ to the reference strain Lb. reuteri DSM20016.*

## Results and Discussion

### *L. reuteri* Isolation and Typing

In the chicken GIT, *L. reuteri* forms biofilm in the crop and is among the most abundant *Lactobacillus* species commonly found in the cecum and colon. However, to date, only seven chicken *L. reuteri* strains (P43, An71, An166, 1366, JCM 1081, CSF8, and SKK-OGDONS-01) have been sequenced, and some of them included in previous phylogenetic analysis (Oh et al., 2010; Frese et al., 2011; Wegmann et al., 2015; Duar et al., 2017; Lee et al., 2017).

By applying a reuterin selective colourimetric method on the plate, a total of 70 *L. reuteri* strains were isolated from feces and cecum of 6 chicken and from the crops of chicken obtained from the abattoir. Among them, 50 were reuterin-positive while 20 were reuterin-negative. The identity of all strains was confirmed by sequencing of the 16S rRNA gene with 100% similar to the reference strain *L. reuteri* DSM20016. The colourimetric method applied allowed an easy phenotypic isolation of reuterin-positive colonies, that accounted for approximately 50% of the bacterial colonies obtained on MRS isolation plates. This result indicated the high frequency of reuterin-producing *L. reuteri* strains in the chicken GIT, compared to the absence of reuterin production phenotype for isolated rodent strains (Walter et al., 2011).

Based on the similarity obtained from ERIC PCR profiles, 31 isolates with unique ERIC profiles were visually selected and further clustered with Bionumerics to confirm the uniqueness (Supplementary Figure S1). Based on the dendrogram obtained for the 31 isolates, 23 reuterin-positive strains with unique ERIC profiles and 2 reuterin-negative strains were selected for whole genome sequencing and further functional characterization (Supplementary Figure S1). Among them, 13 were isolated from cecum and 10 from feces while the 2 reuterin-negative strains originated from the crop.

### Genomes Analysis of New *L. reuteri* Chicken Isolates

The whole genomes of 25 *L. reuteri* strains were sequenced, and draft genomes were characterized. Seven genomes from chicken *L. reuteri* isolates were available prior to this study. The 25 new *L. reuteri* chicken isolates obtained in this study significantly increase the proportion of chicken strain in the *L. reuteri* genome database (*n* = 184).

Strains had an average genome size of 2.159 ± 0.17 Mbp (Table 1). BUSCO assembly assessment showed good quality of assembly with 431 to 433 single-copy orthologous on a total of 433 (Supplementary Figure S2). Three genomes appeared to have lower quality (PTA5_C6B, PTA6_F4, and PTA6_F6), this was reflected by higher number of contigs, bigger genome size, and higher number of CDS compared to the other genomes (Table 1 and Supplementary Figure S3b). Shotgun reads were assembled into contigs higher than 500 bp, ranging from 68 (PTA4_C4) to 657 (PTA5_C6B). The average N50 value, defined as the minimum contig length needed to cover 50% of the genome, was 38337 ± 10.6 while the average guanine-cytosine (GC) content was 38.1 ± 0.11%. The total number of coding sequences (CDs) ranged from 1909 to 2298, depending on the isolates. In every draft genome, 1 tmRNA gene, 45 to 68 tRNA genes and 3 to 6 rRNA genes were identified (Table 1). A total number of 2078 genes was annotated, with a core set of 817 shared genes among the 25 new chicken isolates. The number of annotated genes that were unique to each strain, as compared to the other newly annotated strains, ranged from 0 to 31 (data not shown), showing a similar genetic content of the new chicken isolates of *L. reuteri*. The genomic features of *L. reuteri* isolates of this study in terms of genome size, GC content and number of CDS were comparable with that of the 40 *L. reuteri* NCBI analyzed genomes from different hosts, suggesting not genetic diversity driven by the host (Supplementary Figure S3).

### Genomes of Strains Isolated From Crop and Cecum/Feces Differ in Composition

**FIGURE 1:**
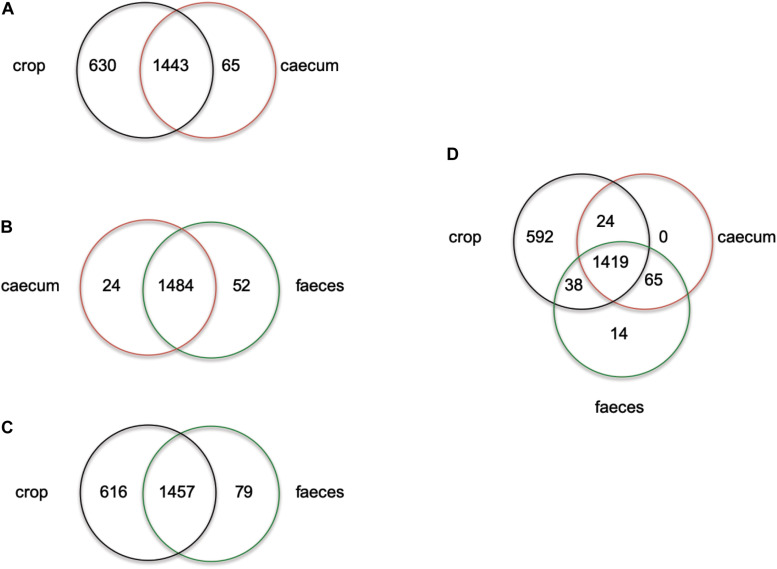
Venn diagram showing the number of unique or shared genes between *L. reuteri c*hicken isolates from crop vs. cecum **(A)**, cecum vs. feces **(B)**, crop vs. feces **(C)** and cecum vs. feces vs. crop **(D)**. Individual unique genes of crop isolates, in each comparison, are listed in Supplementary Table S2 in alphabetic order.

ERIC profiles did not distinguish isolates from feces and cecum this could be due to the transition of strains in the chicken GIT from the cecum to feces. On the other hand, non-reuterin producing *L. reuteri* isolates from the crop (PTA8_1, PTA14_19, PTA5_11) appeared to have a unique ERIC profile (Supplementary Figure S1). To further investigate the ecology of isolates originating from different regions of the chicken GIT, we compared the number of unique and shared genes of isolates from cecum, feces or crop (Figure 1 and Supplementary Table S2). When comparing cecum and feces isolates, few unique genes were identified for cecum isolates, as compared to feces isolates (Figure 1B). Despite a high number of shared genes between crop and cecum (Figure 1A), and crop and feces isolates (Figure 1C), 630 and 616 genes appeared to be unique in crop isolates as compared to cecum and feces, respectively (Figures 1A,C and Supplementary Table S2). When isolates originating from the three GIT regions were compared, 592 genes appeared unique to crop strains (Figure 1D). Indeed, some of the genes unique to strains obtained from the chicken crop include enzymes related arginine, glutaminase, and glutathione metabolism (Supplementary Table S2). These genes were previously shown to support acid resistance in *L. reuteri* (Teixeira et al., 2014), and were upregulated in murine forestomach biofilms compared to gene expression in the lumen of colon and cecum (Schwab et al., 2014). Interestingly, citrate lyase required for citrate metabolism was present only in crop isolates of *L. reuteri.* The optimum pH for citrate metabolism lay between 4.0 to 5.0 (Palles et al., 1998) which is the pH usually measured in the crop of chicken (Kierończyk et al., 2016). Our observations might suggest that there are two populations of *L. reuteri* in the chicken GIT, one inhabiting the crop and the other in the cecum recovered in the feces. A more robust selection and genomes sequencing of strains from chicken crop and cecum is required to test the adaptation of *L. reuteri* to specific location in the chicken GIT.”

### Comparative Phylogenetic Analysis of *L. reuteri* Strains From Different Hosts

The apparent relatedness between microbial community composition in the gut and host phylogeny has been interpreted as evidence of coevolution (Ley et al., 2008). Symbiotic gut microbes associated with the host are predicted to evolve host-specific traits and, as a result, display enhanced ecological performances in their host (Garcia and Gerardo, 2014; Duar et al., 2017). To assess evolution and adaptation of *L. reuteri* strains to different hosts, the gene content of chicken isolates was analyzed together with that of 40 *L. reuteri* strains available by NCBI, obtained from different hosts: human (6), rat (1), mouse (3), pig (4), sourdough (4), goat (5), sheep (4), cow (4), horse (3) and chicken (6).

**FIGURE 2:**
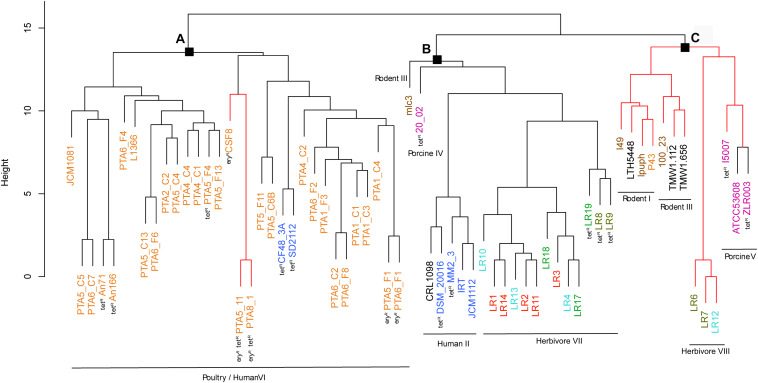
Phylogenetic tree based on gene content matrix (presence or absence of annotated gene) of 65 *L. reuteri* strains from different hosts (25 genomes from this study and 40 from NCBI, Supplementary Table S1). Different colors represent different hosts, blue: human; yellow: chicken; pink: pig; brown: mouse/rat; light blue: cow; red: goat; green: horse; brown/green: sheep; black: sourdough. The red branches indicate the reuterin-negative strains. **(A–C)** indicate identified clusters. Strains which harbor AMR genes for tetracycline (tet) and erythromycin (ery) are indicated as tetR and eryR, respectively.

The gene content tree, in which strains sharing more genes clustered together, identified three main clusters namely cluster A, cluster B, and cluster C, that contained previously observed *L. reuteri* lineages (Oh et al., 2010) (Figure 2 and Supplementary Table S1). Those host-adapted lineages were first described after the characterization of the genetic structure of *L. reuteri* strains isolated from human, mouse, rat, pig, chicken, and turkey, and the same lineage names were also applied in our study for coherency (Oh et al., 2010). Regarding herbivore strains, no lineages have been defined before our study. Cluster A, corresponding to the previously defined poultry/human lineage VI, comprised all 25 *L. reuteri* chicken isolates of this study and all, except one (P43), chicken NCBI isolates (Figure 2). The same cluster also included two human strains (SD2112 and CF48-3A). Those two isolates were previously described as clustering in an unexpected way (Oh et al., 2010; Duar et al., 2017; Lee et al., 2017). Identified cluster B included the majority of herbivorous isolates (new defined herbivorous lineage VII of our study) in addition to four human (DSM20016, MM2_3, IRT and JCM1112), one sourdough (CRL1098), one rodent (mlc3) and one porcine (20-02) strains, belonging to lineages II, III and IV previously defined (Oh et al., 2010). Cluster C was composed of pig, herbivorous, sourdough, and rodent isolates (Figure 2) corresponding to lineages I, III, V and newly herbivorous VIII lineage. ANI analysis of the 65 *L. reuteri* isolates (Supplementary Figure S4) identified the same three clusters, except strains 20_02 (pig) and mlc3 (mouse) that were assigned to cluster C instead of cluster B.

**FIGURE 3:**
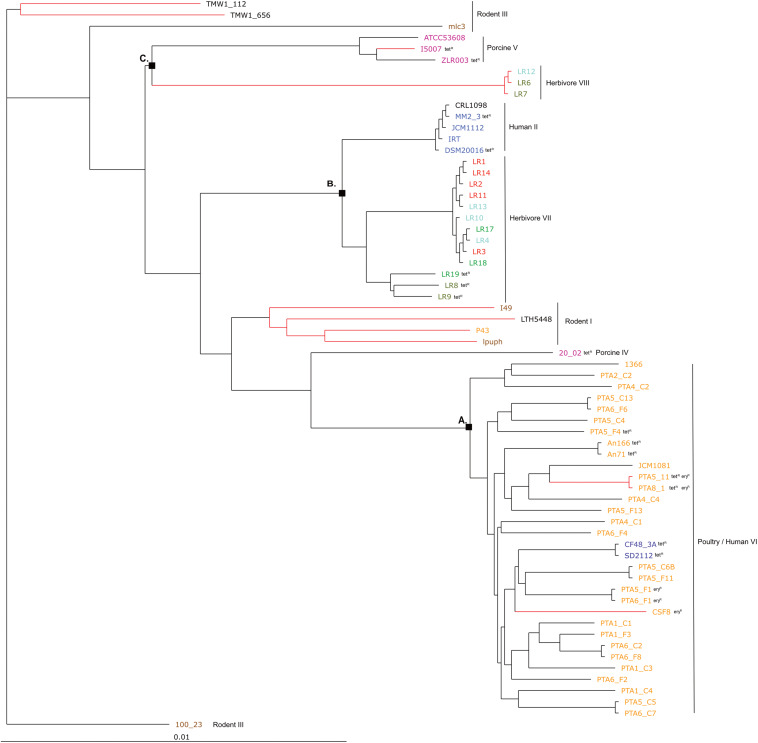
Phylogenetic tree based on core genes of 65 *L. reuteri* strains from different hosts (25 genomes from this study and 40 from NCBI, Supplementary Table S1) based on the neighbor-joining method (PHYLIP implementation). Different colors represent different hosts, blue: human; yellow: chicken; pink: pig; brown: mouse/rat; light blue: cow; red: goat; green: horse; brown/green: sheep; black: sourdough. The red branches indicate the reuterin-negative strains. A, B and C indicate identified clusters in the gene content tree (Figure 1). Strains which harbor AMR genes for tetracycline (tet) and erythromycin (ery) are indicated as tetR and eryR, respectively.

The phylogenetic tree based on core genomes of the 65 genomes covered a core of 1152 genes per genome, for a total of 74880 genes. In agreement with the gene content tree, all isolated *L. reuteri* chicken strains clustered together with NCBI chicken isolates (strains JCM1081, CSF8, An71 and An166), except P43, forming poultry/human lineage VI (Oh et al., 2010). As indicated above, this cluster also included the two human isolates CF48-3A and SD2112 (cluster A, Figure 3). Cluster B was composed of strains from human lineage II and herbivorous lineage VII, as previously above in gene content tree, and the same applied for cluster C which was composed of porcine lineage V and herbivorous lineage VIII. *L. reuteri* strains belonging to rodent lineage III, rodent lineage I and porcine lineage IV clustered differently from the gene content tree, which includes accessory genes. This might indicate gene loss or acquisition of genes by horizontal gene transfer or be just the results from genome sequence quality.

**FIGURE 4:**
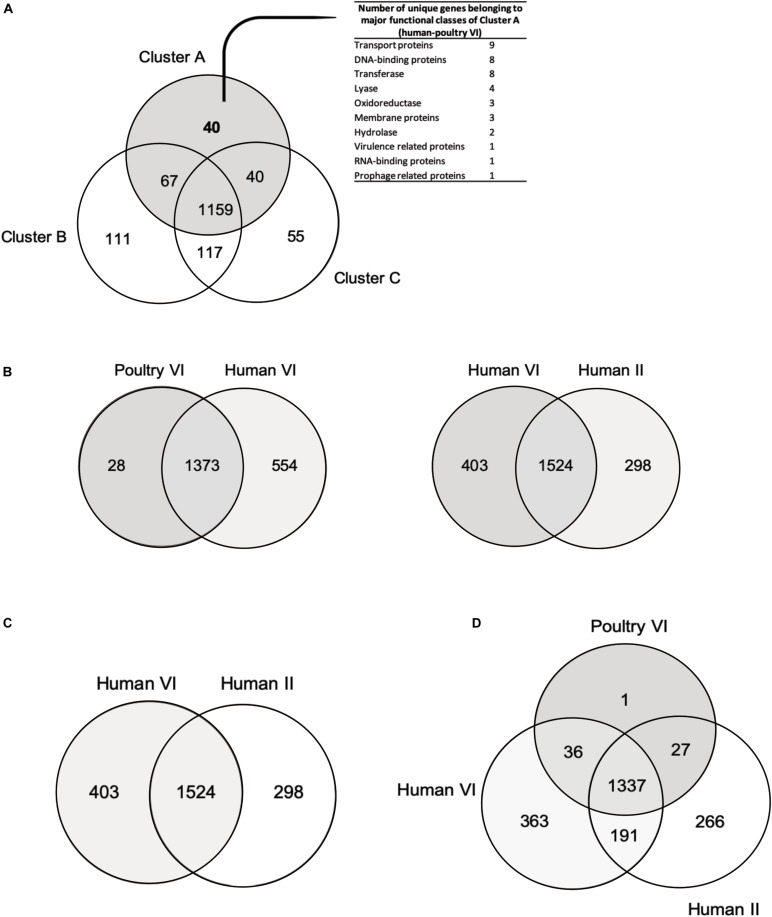
Venn diagrams showing number of reciprocal best hits among different *L. reuteri* subset of core genomes. **(A)** Number of shared and unique genes of core genomes of cluster A (poultry/human lineage VI), cluster B (lineages human II, herbivore VII, porcine IV and rodent III) and cluster C (lineages rodent I, rodent III, herbivore VIII and porcine V). **(B)** Number of shared and unique genes of poultry VI genomes and human VI genomes. **(C)** Number of shared and unique genes between human VI and human II strains. **(D)** Number of shared and unique between poultry VI, human VI and human II lineages. Unique genes of human/poultry VI clades **(A)** and human VI isolates **(B)** are listed in Supplementary Table S3.

Overall, the three different analyses (gene content tree, ANI analysis and core genome tree) performed in this study grouped together human and poultry isolates of lineage VI similar to Frese et al. (2011) (cluster A), human strains of lineage II with herbivore strains of lineage VII strains (cluster B) and porcine strains of lineage V with herbivore strains of lineage VIII strains (cluster C). A previous study demonstrated that isolates of human lineage VI only colonized the chicken and not the human GIT (Duar et al., 2017). When the number of shared or unique genes among poultry lineage VI isolates or human lineage VI isolates were compared, 554 genes appeared to be unique in human VI isolates, compared to only 28 unique genes for chicken isolates (Figure 4B). Furthermore, when comparing human VI with human II isolates (Figure 4C), a higher number of shared genes was shown, and this was also the case when comparing poultry lineage VI with human lineage VI and human lineage II strains (Figure 4D). All analyses performed confirmed the existence and composition of the poultry/human lineage VI, which was substantially enriched by the 25 chicken isolates from our study. Those strains appear to share both core and accessory genes and to be highly similar also at the nucleotide level.

### Genes Unique for the Poultry/Human Lineage VI

Presence of host-specific lineages in itself does not necessarily provide evidence for natural selection, as a cluster can arise by neutral processes, such as genetic dispersion (Oh et al., 2010). It has been demonstrated how strains from rodent display elevated fitness in mice, and biofilm formation in the forestomach is restricted to strains from rodent lineages. Moreover, *L. reuteri* rodents strains were able to effectively colonize rodent host *in vivo* (Oh et al., 2010; Frese et al., 2011; Duar et al., 2017). However, this was not the case for pig isolates (Wegmann et al., 2015; Duar et al., 2017). Here, forty unique genes of the poultry/human lineage VI were identified (Figure 4A and Supplementary Table S3) and were mainly categorized as transport proteins DNA-binding proteins and transferase proteins. Such genes could not be directly linked with adaptation to chicken physiology or feeding. Further studies are needed to elucidate the specific role(s) of those unique genes that may be linked to chicken adaptation.

### Reuterin Synthesis

**FIGURE 5:**
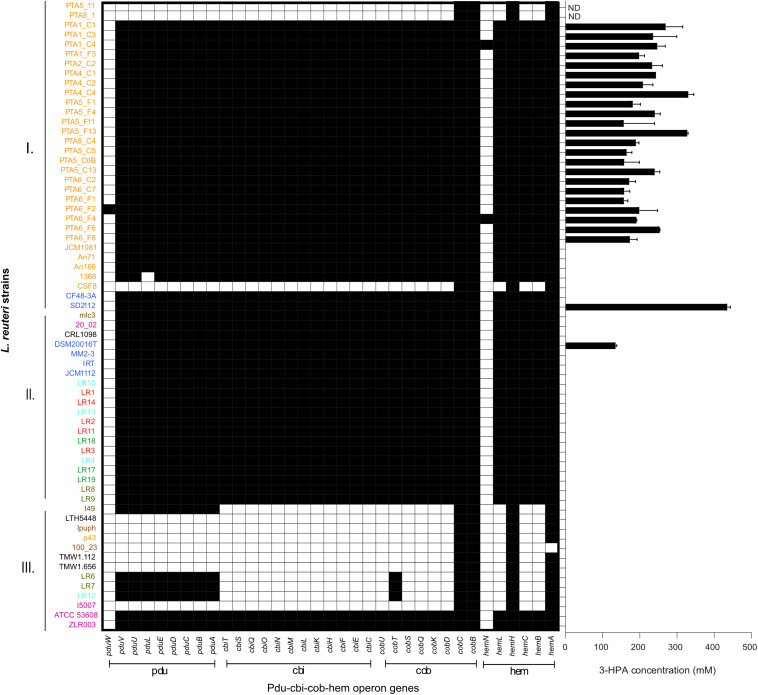
Reuterin operon genes (*pdu-cbi-cob-hem*) detected in the genomes of 65 *L. reuteri* listed in Supplementary Table S1 and 3-HPA production of 25 strains isolated from chicken in this study. Black indicates the presence of a gene. Different colors represent different hosts, blue: human; yellow: chicken; pink: pig; brown: mouse/rat; light blue: cow; red: goat; green: horse; brown/green: sheep; black: sourdough. ND not determined.

The presence and composition of reuterin operon genes (*pdu-cbi-cob-hem*) was investigated in all 65 genomes (Figure 5), and reuterin production was determined as a marker for PduCDE activity. All *L. reuteri* strains isolated in this study that possessed the complete *pdu-cbi-cob-hem* operon produced 3-HPA when incubated in 600 mM glycerol. In contrast, strains, PTA5_11 and PTA8_1 only possessed *hemH, hemA*, *cobC* and *cobB* but lacked all the other operon genes (Figure 5) and therefore did not produce 3-HPA. Under the conditions of the test, 3-HPA yield ranged from 156.9 mM ± 11.0 (PT6_F1) to 330.2 mM ± 14.9 (PTA4_C4) starting from 600 mM glycerol (Figure 5). Reference strains DSM 20016 (human lineage II) produced 132.8 ± 4.3 mM while SD2112 (human lineage VI) produced 432.9 ± 9 0 mM 3-HPA.

All cluster A strains (corresponding to poultry/human lineage VI) harbored *pdu-cob-cbi-hem* genes, except for a small sub-cluster of three by reuterin-negative chicken isolates CSF8, PTA5_11 and PTA8_1. Isolates assigned to Cluster B possessed *pdu-cob-cbi-hem* and have been shown to mostly form reuterin on MRS agar plates overlaid with 500 mM glycerol agar (Walter et al., 2011). Isolates of this cluster lacked *pduW* and *hemN* genes, which therefore seem not essential for reuterin production. These genes were also not detected in the vast majority of reuterin-positive strains isolated in our study (Figure 5). In contrast, the prevalence of *pdu-cob-cbi-hem* scattered in Cluster C comprising isolates of rodent lineages I and III, herbivorous and porcine lineages VIII and V, respectively. Only strains ATCC53608 and ZLR003 possessed a complete functional *pdu-cob-cbi-hem* operon while cluster C rodent isolates lpuph and 100_23 lack the majority of the operon genes.

**FIGURE 6:**
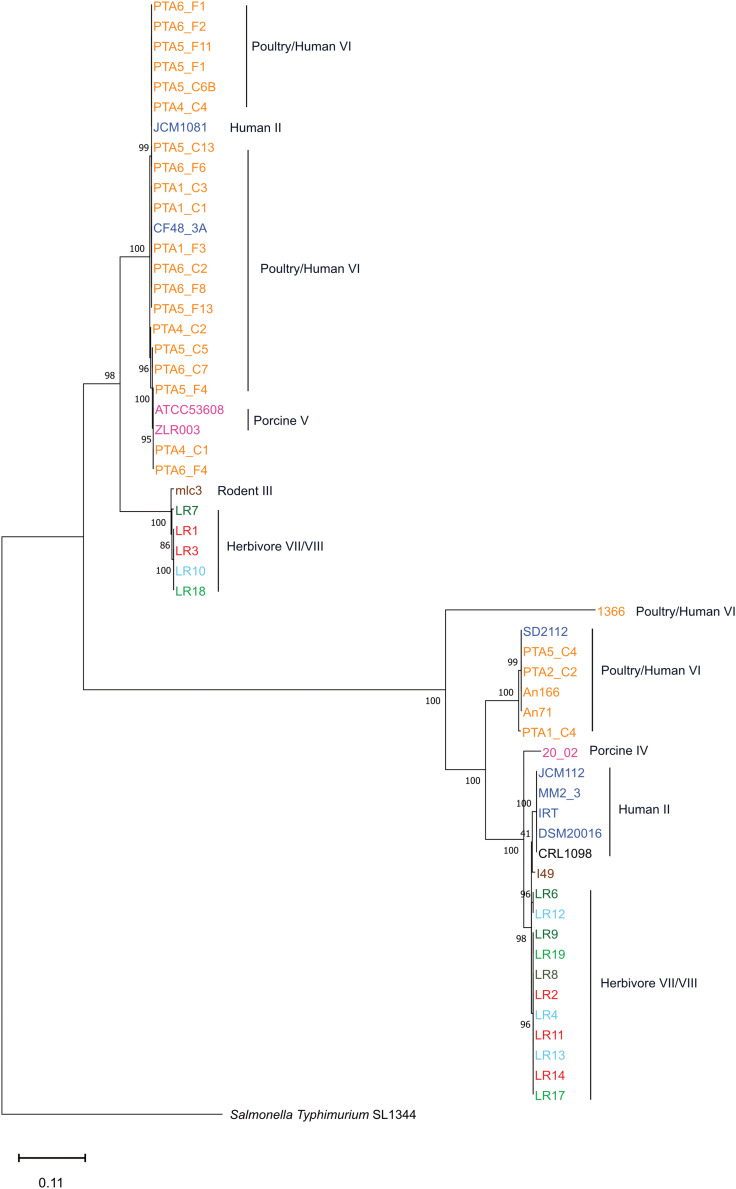
Gene tree based on the nucleotide sequence of concatenated *pduA*, *pduB*, *pduC*, *pduD*, *pduE* genes of *L. reuteri* reuterin-positive genomes of this study (Neighbor Joining Method). Different color represents different hosts, blue: human; yellow: chicken; pink: pig; brown: mouse/rat; light blue: cow; red: goat; green: horse; brown/green: sheep; black: sourdough.

Interestingly, herbivore isolates LR6, LR7, LR12 and the rodent isolate I49 possessed the *pdu* but not the *cbi* and *cob* genes, although cobalamin (*cbi* and *cob* gene) is a cofactor for 3-HPA production. Therefore these strains are likely not able to form 3-HPA from glycerol (or propanal from 1,2-propanediol) unless they acquire the vitamin from other sources or microbes. When looking at the *pduABCDE* phylogenetic tree, LR6, LR12 and I49 are clustered all together, indicating a highly conserved pdu gene cluster (Figure 6).

Rodent strains, which are mostly reuterin-negative based on the analysis of operon genes in this study, are considered the root of the evolutionary history of *L. reuteri*-host associates (Duar et al., 2017), which as a consequence, suggests that the *pdu-cbi-cob-hem* operon and thus reuterin production was acquired later during *L. reuteri*. To further study development of the *pdu* cluster, concatenated sequences of *pduABCDE* were aligned, and a gene tree was constructed (Figure 6). The *pduABCDE* gene tree separated into two different branches each mainly composed of herbivores or poultry/human isolates, not showing clear host-dependent separation (Figure 6). The same clustering was obtained when gene trees were built based on individual *pdu*A-*pduE* genes (Supplementary Figure S5).

### Antimicrobial Susceptibility Profiles of *L. reuteri* Strains

The horizontal transfer of AMR genes is a rising risk concern, and the absence of transferable AMR genes must be demonstrated for application of new strains in food and feed (Rychen et al., 2018). Antimicrobials used in farmed animals for diseases prevention have been associated with an increase frequency of resistant bacteria in chickens, swine, and other food-producing animals GIT (Chang et al., 2015). The high use of antimicrobials in animal production is likely to accelerate the development of AMR in pathogens, as well as in commensal organisms, resulting in treatment failures, economic losses and source of the gene pool for transmission to humans (Chang et al., 2015). Chicken is one of the most widespread food industries worldwide, and various antimicrobials are used to treat infections mainly in chicks (Sahoo et al., 2010; Agyare et al., 2019).

The antimicrobial susceptibility profile of *L. reuteri* chicken isolates and reference strains DSM20016 and SD2112 showed that all strains were sensitive to cefotaxime (MIC values from 0.016 to 1 μg/mL) (Table 2). All chicken isolates were also sensitive to penicillin with MIC values from 0.02 and 3 μg/mL, in contrast to DSM20116 and SD2112 that showed resistance to this antibiotic (MIC > 256 ug/mL). Penicillin resistance was shown to result from point mutations of the chromosomally located genes encoding penicillin-binding proteins Pbp (Rosander et al., 2008). Penicillin-binding genes *pbpX*, *pbpF*, *pbpB* and *ponA* were identified in all 65 strains with both resistant and sensitive phenotype (Table 2 and Supplementary Table S4). Several SNPs were observed for DSM20016 (Table 3), especially in *ponA* and *pbpX_2*, which led to point mutations of the corresponding proteins. Only one amino acid substitution at position 134 of PbpX_2 was shared among the two resistant strains: DSM20016 possessed a Q instead of H (H_134_Q) while in the same position strain SD2112 had a Y (H_134_Y). The substitution at this position may be the one responsible for the penicillin-resistant phenotype observed for DSM20016 and SD2112.

**TABLE 2:** Antimicrobial resistance (AMR) profiles of 25 *L. reuteri* strains isolated from chicken in this study and of reference strains measured using MTS^TM^ strips (MIC, μg/mL), and associated AMR genes detected in their draft genomes.

Strain	Origin	CFX	ERM		PEN		TET		VAN	CIP	
						
		MIC	Genotype	MIC	Genotype	MIC	Genotype	MIC	MIC	Genotype	MIC
PTA5_11	Chicken	0.38	*erm*(B)^1^	>256	*ponA, pbpX, pbpF, pbpB*	3	*tetA, tetO, tetW*^1^	>256	>256	*gyrA, gyrB, parB, parC, parE, prmA, prmC*	>32
PTA8_1	Chicken	1	*erm*(B)^1^	>256	*ponA, pbpX, pbpF, pbpB*	2	*tetA, tetO, tetW*^1^	>256	>256	*gyrA, gyrB, parB, parC, parE, prmA, prmC*	>32
PTA1_C1	Chicken	0.25		2	*ponA, pbpX, pbpF, pbpB*	2	*tetA, tetO*	12	>256	*gyrA, gyrB, parB, parC, parE, prmA, prmC*	>32
PTA1_C3	Chicken	0.023		2	*ponA, pbpX, pbpF, pbpB*	0.38	*tetA, tetO*	6	>256	*gyrA, gyrB, parB, parC, parE, prmA, prmC*	>32
PTA1_C4	Chicken	0.38		2	*ponA, pbpX, pbpF, pbpB*	3	*tetA, tetO*	8	>256	*gyrA, gyrB, parB, parC, parE, prmA, prmC*	>32
PTA1_F3	Chicken	0.25		8	*ponA, pbpX, pbpF, pbpB*	1.5	*tetA, tetO*	16	>256	*gyrA, gyrB, parB, parC, parE, prmA, prmC*	>32
PTA2_C2	Chicken	0.016		1.5	*ponA, pbpX, pbpF, pbpB*	1.5	*tetA, tetO*	12	>256	*gyrA, gyrB, parB, parC, parE, prmA, prmC*	>32
PTA4_C1	Chicken	0.016		2	*ponA, pbpX, pbpF, pbpB*	0.5	*tetA, tetO*	12	>256	*gyrB, parB, parC, parE, prmA, prmC*	>32
PTA4_C2	Chicken	0.016		1	*ponA, pbpX, pbpF, pbpB*	3	*tetA, tetO*	24	>256	*gyrA, gyrB, parB, parC, parE, prmA, prmC*	>32
PTA4_C4	Chicken	0.016		1.5	*ponA, pbpX, pbpF, pbpB*	0.094	*tetA, tetO*	6	>256	*gyrA, gyrB, parB, parC, parE, prmA, prmC*	>32
PTA5_F1	Chicken	0.064	*erm*(B)^1^	>256	*ponA, pbpX, pbpF, pbpB*	3	*tetA, tetO*	12	>256	*gyrA, gyrB, parB, parC, parE, prmA, prmC*	>32
PTA5_F4	Chicken	0.032		3	*ponA, pbpX, pbpF, pbpB*	0.5	*tetA, tetO, tetW*^1^	>256	>256	*gyrA, gyrB, parB, parC, parE, prmA, prmC*	>32
PTA4_F11	Chicken	0.016		4	*ponA, pbpX, pbpF, pbpB*	3	*tetA, tetO*	6	> 256	*gyrA, gyrB, parB, parC, parE, prmA, prmC*	>32
PTA5_F13	Chicken	0.016		3	*ponA, pbpX, pbpF, pbpB*	0.75	*tetA, tetO*	8	>256	*gyrA, gyrB, parB, parC, parE, prmA, prmC*	>32
PTA5_C4	Chicken	0.094		2	*ponA, pbpX, pbpF, pbpB*	2	*tetA, tetO*	16	>256	*gyrA, gyrB, parB, parC, parE, prmA, prmC*	>32
PTA5_C5	Chicken	0.25		1.5	*ponA, pbpX, pbpF, pbpB*	1.5	*tetA, tetO*	4	>256	*gyrA, gyrB, parB, parC, parE, prmA, prmC*	>32
PTA5_C6B	Chicken	0.47		2	*ponA, pbpX, pbpF, pbpB*	2	*tetA, tetO*	12	>256	*gyrA, gyrB, parB, parC, parE, prmA, prmC*	>32
PTA5_C13	Chicken	0.016		3	*ponA, pbpX, pbpF, pbpB*	4	*tetA, tetO*	6	>256	*gyrA, gyrB, parB, parC, parE, prmA, prmC*	>32
PTA6_C2	Chicken	0.016		4	*ponA, pbpX, pbpF, pbpB*	0.5	*tetA, tetO*	8	>256	*gyrA, gyrB, parB, parC, parE, prmA, prmC*	>32
PTA6_C7	Chicken	0.19		1.5	*ponA, pbpX, pbpF, pbpB*	0.023	*tetA, tetO*	16	>256	*gyrA, gyrB, parB, parC, parE, prmA, prmC*	>32
PTA6_F1	Chicken	0.016	*erm*(B)^1^	>256	*ponA, pbpX, pbpF, pbpB*	4	*tetA, tetO*	16	>256	*gyrA, gyrB, parB, parC, parE, prmA, prmC*	>32
PTA6_F2	Chicken	0.38		0.016	*ponA, pbpX, pbpF, pbpB*	0.5	*tetA, tetO*	2	>256	*gyrA, gyrB, parB, parC, parE, prmA, prmC*	>32
PTA6_F4	Chicken	0.016		6	*ponA, pbpX, pbpF, pbpB*	0.38	*tetA, tetO*	24	>256	*gyrA, gyrB, parB, parC, parE, prmA, prmC*	>32
PTA6_F6	Chicken	0.023		1.5	*ponA, pbpX, pbpF, pbpB*	3	*tetA, tetO*	1.5	>256	*gyrA, gyrB, parB, parC, parE, prmA, prmC*	>32
PTA6_F8	Chicken	0.19		8	*ponA, pbpX, pbpF, pbpB*	0.75	*tetA, tetO*	16	>256	*gyrA, gyrB, parB, parC, parE, prmA, prmC*	>32
DSM20016	Human	0.38		1.5	ponA, pbpX, pbpF, pbpB*	>256	tetA, tetO, tetW **	>256	>256	*gyrA, gyrB, parB, parC, parE, prmA, prmC*	>32
SD2112	Human	0.25		0.125	ponA, pbpX, pbpF, pbpB*	>256	tetA, tetO, tetW***	>256	>256	*gyrA, gyrB, parB, parC, parE, prmA, prmC*	>32

*^∗^Mutations in penicillin-binding proteins of strain DSM20116 and SD2112 are shown in Table 3. ^∗∗^tetW detected on plasmid_SD2112_pLR581. ^∗∗∗^tetW detected by PCR. ^1^AMR genes deduced to be allocated on plasmid, see Supplementary Table S5. CFX, cefotaxime; ERM, erythromycin; PEN, penicillin; TET, tetracycline; VAN; vancomycin; CIP, ciprofloxacin. MIC, minimum inhibitory concentration.*

**TABLE 3:** Point mutations identified in penicillin-binding protein genes of resistant strains *L. reuteri* DSM20016 and *L. reuteri* SD2112.

Strain	Gene	Mutation(s) in penicillin-binding proteins
DSM20016	*ponA*	D_322_N / S_537_A / V_538_A / D_712_E / A_723_V / S_725_N/ A_740_T / D_41_1V / Y_412_F / Q_491_L
	*pbpB*	A_361_T / H_495_Q / T_586_P / V_613_A / L_714_S
	*pbpF*	D_9_G / T_22_A
	*pbpX*_1	V_26_I / T_61_P / I_62_V / A_63_T / K_100_R / D_123_E
	*pbpX*_2	V_19_I / L_26_V / H_30_R / I_49_L / V_86_I / D_96_N / T_111_A / N_127_T / K_132_R / **H_134_Q** / R_141_H / T_170_A / R_185_N / Q_187_H / K_244_N / T_252_I / L_255_F / Y_320_F / D_322_A / K_333_T
SD2112	*ponA*	D_411_V / Y_412_F / Q_491_L
	*pbpX*_1	R_141_H
	*pbpX*_2	E_124_K / **H_134_Y**
	*pbpX*_3	P_57_L / L_193_P

*Amino acids changes in the resistant strains, compared to all other 25 L. reuteri sensitive strains, are indicated. In bold, a shared mutation for both resistance strain with a different amino acids substitution.*

Four chicken isolates of this study (PTA5_11, PTA8_1, PTA5_F1 and PTA6_F1) showed resistance phenotype (MIC > 256 μg/mL) to erythromycin, confirmed by the presence of *ermB*, which is usually found on a plasmid (Egervärn et al., 2009; Abriouel et al., 2015). The *ermB* gene encodes enzymes that modify the 23S rRNA by adding one or two methyl groups, reducing the binding to the ribosome of different classes of antibiotics (Gupta et al., 2003). The presence or absence of *ermB* gene in the genome of erythromycin resistant strains was confirmed by using PCR (data not shown). For all resistant strains (PTA5_11, PTA8_1, PTA5_F1, and PTA6_F1), *ermB* appeared to be mostly likely located on plasmid (Supplementary Table S5). Among the 40 NCBI strains analyzed in this study, e*rmB* was also detected in the genome of chicken isolate CSF8 (Figures 2, 3).

Tetracycline resistance genes *tetA* and *tetO* were detected in all 65 *L. reuteri* genomes analyzed but did not appear to be directly correlated with this resistance phenotype (Table 2 and Supplementary Table S4). The only 3 chicken isolates resistant to tetracycline (MIC > 256 μg/mL) possessed additionally the *tetW* in their genome (PTA5_11, PTA8_1, and PTA5_F4), similar to DSM20016 and SD2112 (MIC > 256 μg/mL). Tetracycline resistance has been described to be located on a plasmid, for example, in SD2112 (pLR581) (Kastner et al., 2006). For DSM20016, *tetW* was not detected on the chromosome but was amplified by PCR. For all resistant chicken strains of this study (PTA5_11, PTA8_1, and PTA5_F4), *tetW* gene was deduced to be plasmid located (Supplementary Table S5). Besides *tetW*, *tetM, tetL* and *tetC* were also associated with tetracycline resistance were identified in the genomes of different isolates (MM2_3, I5007, 20_02, ZLR003, LR8, LR9, and LR19). Interestingly, tetracycline-resistant chicken isolates (An71, An166) harbored *tetW* while herbivorous resistant strains (LR8, LR9, and LR19) possessed *tetM*. In contrast, pig isolates harbored *tetM* and *tetW* (I5007 and 20_02) or *tetW* and *tetL* (ZLR003) (Supplementary Table S4). Tetracyclines are widespread antimicrobials extensively used in livestock partly due to their broad-spectrum activity and low cost compared to other antibiotics (Economou and Gousia, 2015; Granados-Chinchilla and Rodríguez, 2017). This might explain the widespread of tetracycline resistance gene pool in farm animals.

Lactobacilli are suggested to be intrinsically resistant to vancomycin and ciprofloxacin (Hummel et al., 2007). In agreement with previous studies (Egervärn et al., 2009), all chicken *L. reuteri* strains of this study were resistant to vancomycin (MIC > 256 μg/mL). Vancomycin resistance in lactobacilli is linked to the *vanX* gene encoding a d-Ala-d-Ala dipeptidase (Klein et al., 2000). Other vancomycin resistance genes were described in the literature with *vanA*, *vanB*, *vanC* and *vanE* (Klein et al., 2000; Kastner et al., 2006). None of those genes was detected in the genomes of *L. reuteri* in agreement with previous studies (Klein et al., 2000; Kastner et al., 2006). However, changes in membrane composition have also been associated with intrinsic vancomycin resistance (Delcour et al., 1999). *VanH*, a D-lactate dehydrogenase gene was detected in one pig strain (ATCC 53608) (Faron et al., 2016). The same gene had been previously associated with vancomycin resistance of *Enterococcus faecium* (Bugg et al., 1991). Ciprofloxacin resistance seems to be widely spread among lactobacilli (Hummel et al., 2007; Li et al., 2015; Guo et al., 2017). All 25 chicken isolates were resistant to ciprofloxacin with MIC > 32 μg/mL, and all 65 genomes analyzed possessed the six genes for which mutations were correlated with ciprofloxacin resistance, namely *gyrB*, *parB*, *parC*, *parE*, *prmA* and *prmC*, while *gyrA* was present in all but one (PTA4_C1) isolate.

*L. reuteri* has been affiliated to different hosts, which might be exposed to different levels and types of antimicrobials and is commercially used as probiotic in food and feed (Hou et al., 2015; Ávila et al., 2017; Asare et al., 2018; Chen et al., 2018). The results of this study indicated that *L. reuteri* chicken isolates harbor some AMR genes. In view of application *L. reuteri* in feed to prevent pathogen infection, strains without transferable AMR genes must be carefully selected. The first applied *L. reuteri* probiotic strains (SD2112) harbors the tetracycline-resistant gene *tetw* on a plasmid (Kastner et al., 2006). This strain was, however, cured for the plasmid-free daughter strain DSM17938 (Rosander et al., 2008), which is commercially used.

## Conclusion

This study substantially enriched the pool of chicken *L. reuteri* strains for comparative genomics and evolutionary studies. Isolates from the crop appeared to have a unique genetic profile compared to isolates from more distal region of the GIT, which might suggest that chicken are colonized by two populations of *L. reuteri*. The phylogenetic analysis confirmed the co-evolution of human isolates of lineage VI with chicken (poultry/human lineage VI). Despite the high number of chicken isolates of lineage VI, the two human isolates still shared a higher number of genes with isolates belonging to human lineage II, as compared to poultry lineage VI. The pool of *L. reuteri* chicken isolates of this study may be useful to select and characterize strains exhibiting reuterin production activity, and possibly develop application in chicken, as a natural antimicrobial system to prevent pathogen infections and colonization of the chicken GIT.

## Data Availability Statement

The datasets generated for this study can be found in the BioProject ID: PRJNA473635.

## Ethics Statement

Swiss regulation did not require the study to be reviewed or approved by an ethics committee because the samples were taken under direct and strict supervision of a veterinarian.

## Author Contributions

AG, PA, CS, RS, and CL designed the study. AG and PA conducted the experiments and analyzed the data. AG, PA, CS, NZ, RG, and CL drafted the manuscript. All authors read and approved the final manuscript.

## Conflict of Interest

The authors declare that the research was conducted in the absence of any commercial or financial relationships that could be construed as a potential conflict of interest.
