# Supplementary material for: Isolation and Comparative Genomic Analysis of Reuterin-Producing Lactobacillus reuteri From the Chicken Gastrointestinal Tract
Source: Front Microbiol. 2020 Jun 4;11:1166. doi: 10.3389/fmicb.2020.01166 (PMC7326114; doi:10.3389/fmicb.2020.01166)
Supplement: Supplementary file 1 [file Data_Sheet_1.docx]

**SUPPLEMENTARY MATERIAL**

**Isolation and comparative genomic analysis of reuterin-producing *Lactobacillus reuteri* from chicken gastrointestinal tract**

Anna Greppi^1 δ^, Paul Tetteh Asare^1 δ^, Clarissa Schwab^1^, Niklaus Zemp^2^, Roger Stephan^3^, Christophe Lacroix^1*^

^1^ Institute of Food, Nutrition and Health. Laboratory of Food Biotechnology, ETH, Zürich

^2^ Genetic Diversity Centre, ETH, Zürich

^3^ Institute for Food Hygiene and Safety, University of Zürich, Zürich, Switzerland

^δ^ The authors equally contributed to the manuscript

* Corresponding author: [christophe.lacroix@hest.ethz.ch](mailto:christophe.lacroix@hest.ethz.ch)

**Supplementary Table S1.** *L. reuteri* strains with genomes published in NCBI used for comparative genomics. Genomes were retrieved from NCBI and annotated with the same pipeline used for the 25 draft genomes of this study.

| ***L. reuteri* strains** | **Host** | **Lineage^2^** | **Country of Origin** | **NCBI accession number** | **Level (N.)^1^** | **Size (Mb)** |
| --- | --- | --- | --- | --- | --- | --- |
| P43 | Chicken | I | USA | MCNS00000000 | Contig (74) | 2.15106 |
| An71 | Chicken | ND | ND | NZ_NFHN00000000 | Contig (119) | 2.28085 |
| An166 | Chicken | ND | ND | NZ_NFKV00000000 | Contig (105) | 2.24803 |
| 1366 | Chicken | VI | Denmark | NZ_NBBG00000000 | Scaffold (93) | 2.06291 |
| JCM 1081 | Chicken | VI | Japan | NZ_NBBD00000000 | Scaffold (61) | 2.31353 |
| CSF8 | Chicken | VI | USA | NZ_NBBE00000000 | Scaffold (107) | 1.95205 |
| DSM20016 | Human | II | Germany | NC_009513 | Scaffold (150) | 1.93586 |
| IRT | Human | II | South Korea | NZ_CP011024 | Complete | 1.99397 |
| JCM 1112 | Human | II | Japan | NC_010609 | Complete | 2.03941 |
| CF48_3A | Human | VI | USA | NZ_ACHG00000000.1 | Contig (92) | 2.1079 |
| MM2_3 | Human | II | USA | NZ_ACLB00000000.1 | Contig (95) | 2.01572 |
| SD2112 | Human | VI | Peru | NC_015697 | Complete | 2.31684 |
| 100_23 | Rat | III | New Zealand | AAPZ00000000 | Contig (2) | 2.30556 |
| I49 | Mouse | III | Switzerland | NZ_CP015408 | Complete | 2.04477 |
| mlc3 | Mouse | III | USA | AEAW00000000 | Contig (126) | 2.01863 |
| lpuph | Mouse | I | USA | AEAX00000000 | Contig (127) | 2.11662 |
| ATCC 53608 | Pig | IV | Sweden | NZ_LN906634 | Complete | 2.09124 |
| I5007 | Pig | IV | China | NC_021494 | Complete | 2.09328 |
| ZLR003 | Pig | IV | China | NZ_CP014786 | Complete | 2.2341 |
| 20_02 | Pig | V | Germany | CZDD00000000.1 | Scaffold (188) | 2.23295 |
| CRL1098 | Sourdough | II | Argentina | LYWI00000000 | Contig (45) | 1.96303 |
| TMW1.656 | Sourdough | III | Germany | JOSW00000000 | Contig (17) | 1.94954 |
| TMW1.112 | Sourdough | III | Germany | JOKX00000000 | Contig (12) | 2.03203 |
| LTH5448 | Sourdough | I | Germany | JOOG00000000 | Contig (36) | 1.9803 |
| LR1 | Goat | ND | China | QGID00000000 | Scaffold (171) | 2.2556 |
| LR2 | Goat | ND | China | QGIC00000000 | Scaffold (201) | 2.36754 |
| LR3 | Goat | ND | China | QGIB00000000 | Scaffold (181) | 2.30022 |
| LR11 | Goat | ND | China | QGIA00000000 | Scaffold (204) | 2.36581 |
| LR14 | Goat | ND | China | QGHZ00000000 | Scaffold (163) | 2.25127 |
| LR6 | Sheep | ND | China | QGHY00000000 | Scaffold (379) | 2.13973 |
| LR7 | Sheep | ND | China | QGHX00000000 | Scaffold (364) | 2.13843 |
| LR8 | Sheep | ND | China | QGHW00000000 | Scaffold (216) | 2.25588 |
| LR9 | Sheep | ND | China | QGHV00000000 | Scaffold (230) | 2.30327 |
| LR4 | Cow | ND | China | QGHU00000000 | Scaffold (169) | 2.16644 |
| LR10 | Cow | ND | China | QGHT00000000 | Scaffold (363) | 2.4417 |
| LR12 | Cow | ND | China | QGHS00000000 | Scaffold (391) | 2.15145 |
| LR13 | Cow | ND | China | QGHR00000000 | Scaffold (194) | 2.36533 |
| LR17 | Horse | ND | China | QGHQ00000000 | Scaffold (314) | 2.20569 |
| LR18 | Horse | ND | China | QGHP00000000 | Scaffold (614) | 2.47021 |
| LR19 | Horse | ND | China | QGHO00000000 | Scaffold (225) | 2.20232 |

^1^Levels: complete, scaffold or contig, in parenthesis the number of Scaffolds as indicated in NCBI database.

^2^ Previously defined lineages in the literature (Lee et al., 2017; Wegmann et al., 2015; Zheng et al., 2015; Frese et al., 2011).

n.d. not determined before this study

**Supplementary Table S2.** Unique individual genes in the *L. reuteri* isolates from crop, as compared to caecum and faces isolates, as illustrated in Figure 1.

| **Unique genes of crop isolates** | | |
| --- | --- | --- |
| **Crop vs caecum isolates (n° 630)** | **Crop vs faeces isolates (n° 616)** | **Crop vs faeces and caecum isolates (n°592)** |
| [Citrate [pro-3S]-lyase] ligase | [Citrate [pro-3S]-lyase] ligase | [Citrate [pro-3S]-lyase] ligase |
| 2-(5-triphosphoribosyl)-3-dephosphocoenzyme- A synthase | 2-(5-triphosphoribosyl)-3-dephosphocoenzyme- A synthase | 2-(5-triphosphoribosyl)-3-dephosphocoenzyme- A synthase |
| 2-dehydropantoate 2-reductase (n°2) | 2-dehydropantoate 2-reductase (n°3) | 2-dehydropantoate 2-reductase (n°3) |
| 2-keto-4-pentenoate hydratase (n°2) | 2-keto-4-pentenoate hydratase | 2-keto-4-pentenoate hydratase |
| 3-ketoacyl-(acyl-carrier-protein) reductase |  |  |
| 3-oxoacyl-[acyl-carrier-protein] reductase FabG (n°2) | 3-oxoacyl-[acyl-carrier-protein] reductase FabG (n°2) | 3-oxoacyl-[acyl-carrier-protein] reductase FabG (n°2) |
| 4-phosphopantetheinyl transferase sfp | 4-phosphopantetheinyl transferase sfp | 4-phosphopantetheinyl transferase sfp |
| 5-amino-6-(5-phosphoribosylamino)uracil reductase | 5-amino-6-(5-phosphoribosylamino)uracil reductase | 5-amino-6-(5-phosphoribosylamino)uracil reductase |
| 6,7-dimethyl-8-ribityllumazine synthase | 6,7-dimethyl-8-ribityllumazine synthase | 6,7-dimethyl-8-ribityllumazine synthase |
| AAA-like domain protein | AAA-like domain protein | AAA-like domain protein |
| AB hydrolase superfamily protein YdjP | AB hydrolase superfamily protein YdjP | AB hydrolase superfamily protein YdjP |
| ABC-2 family transporter protein (n°2) | ABC transporter substrate binding protein | ABC-2 family transporter protein (n°2) |
|  | ABC-2 family transporter protein (n°2) |  |
| ABC-type transporter ATP-binding protein EcsA | ABC-type transporter ATP-binding protein EcsA | ABC-type transporter ATP-binding protein EcsA |
| Abi-like protein | Abi-like protein | Abi-like protein |
| Accessory gene regulator protein A | Accessory gene regulator protein A | Accessory gene regulator protein A |
| Accessory Sec system protein Asp2 | Accessory Sec system protein Asp2 | Accessory Sec system protein Asp2 |
| Accessory Sec system protein Asp3 | Accessory Sec system protein Asp3 | Accessory Sec system protein Asp3 |
|  | Acetyltransferase (GNAT) family protein |  |
| Acyltransferase family protein (n°2) | Acyltransferase family protein (n°2) | Acyltransferase family protein (n°2) |
| ADP-ribosylglycohydrolase | ADP-ribosylglycohydrolase | ADP-ribosylglycohydrolase |
| Agglutinin receptor precursor | Agglutinin receptor precursor | Agglutinin receptor precursor |
| Alcohol dehydrogenase 1 | Alcohol dehydrogenase 1 | Alcohol dehydrogenase 1 |
| Amino-acid permease RocE | Amino-acid permease RocE | Amino-acid permease RocE |
| anaerobic benzoate catabolism transcriptional regulator (n°3) | anaerobic benzoate catabolism transcriptional regulator (n°3) | anaerobic benzoate catabolism transcriptional regulator (n°3) |
| Antitoxin MazE | Antitoxin MazE | Antitoxin MazE |
| antitoxin YefM | antitoxin YefM | antitoxin YefM |
| Arabinogalactan endo-1,4-beta-galactosidase precursor | Arabinogalactan endo-1,4-beta-galactosidase precursor | Arabinogalactan endo-1,4-beta-galactosidase precursor |
| Arabinose metabolism transcriptional repressor | Arabinose metabolism transcriptional repressor | Arabinose metabolism transcriptional repressor |
| Archaeal ATPase | Archaeal ATPase | Archaeal ATPase |
| Arginine transport ATP-binding protein ArtM | Arginine transport ATP-binding protein ArtM | Arginine transport ATP-binding protein ArtM |
| Arginine transport system permease protein ArtQ | Arginine transport system permease protein ArtQ | Arginine transport system permease protein ArtQ |
| Arginine/agmatine antiporter | Arginine/agmatine antiporter (n°2) | Arginine/agmatine antiporter |
| Arginine/ornithine antiporter |  | Arginine/ornithine antiporter |
| Arsenate-mycothiol transferase ArsC2 | Arsenate-mycothiol transferase ArsC2 | Arsenate-mycothiol transferase ArsC2 |
| Aryl-alcohol dehydrogenase | Aryl-alcohol dehydrogenase | Aryl-alcohol dehydrogenase |
| ATP-dependent Clp protease ATP-binding subunit ClpC | ATP-dependent Clp protease ATP-binding subunit ClpC | ATP-dependent Clp protease ATP-binding subunit ClpC |
| beta-1,6-galactofuranosyltransferase | beta-1,6-galactofuranosyltransferase | beta-1,6-galactofuranosyltransferase |
| Beta-1,6-galactofuranosyltransferase WbbI (n°2) | Beta-1,6-galactofuranosyltransferase WbbI (n°2) | Beta-1,6-galactofuranosyltransferase WbbI (n°2) |
| Beta-galactosidase LacZ | Beta-galactosidase LacZ | Beta-galactosidase LacZ |
| Bifunctional autolysin precursor | Beta-monoglucosyldiacylglycerol synthase | Bifunctional autolysin precursor |
|  | Bifunctional autolysin precursor |  |
| Brinker DNA-binding domain protein | Brinker DNA-binding domain protein | Brinker DNA-binding domain protein |
| CAAX amino terminal protease self- immunity | CAAX amino terminal protease self- immunity | CAAX amino terminal protease self- immunity |
| carbamoyl phosphate synthase small subunit | carbamoyl phosphate synthase small subunit | carbamoyl phosphate synthase small subunit |
| Carbohydrate diacid regulator | Carbohydrate diacid regulator |  |
| Caudovirus prohead protease | Caudovirus prohead protease | Caudovirus prohead protease |
| Chain length determinant protein | Chain length determinant protein | Chain length determinant protein |
| Chromosome partition protein Smc (n°4) | Chromosome partition protein Smc (n°5) | Chromosome partition protein Smc (n°5) |
| Chromosome-partitioning ATPase Soj | Chromosome-partitioning ATPase Soj | Chromosome-partitioning ATPase Soj |
| Citrate lyase acyl carrier protein | Citrate lyase acyl carrier protein | Citrate lyase acyl carrier protein |
| Citrate lyase alpha chain | Citrate lyase alpha chain | Citrate lyase alpha chain |
| Citrate lyase subunit beta | Citrate lyase subunit beta | Citrate lyase subunit beta |
| Competence protein | Competence protein | Competence protein |
|  | Copper chaperone CopZ |  |
| Core-2/I-Branching enzyme | Core-2/I-Branching enzyme | Core-2/I-Branching enzyme |
|  | Cyclic pyranopterin monophosphate synthase |  |
| Cyclic pyranopterin monophosphate synthase accessory protein | Cyclic pyranopterin monophosphate synthase accessory protein | Cyclic pyranopterin monophosphate synthase accessory protein |
| Cystine-binding periplasmic protein precursor | Cystine-binding periplasmic protein precursor | Cystine-binding periplasmic protein precursor |
| cytosine permease |  |  |
| Deoxyribose-phosphate aldolase | Deoxyribose-phosphate aldolase |  |
| Deoxyribose-phosphate aldolase 1 | Deoxyribose-phosphate aldolase 1 | Deoxyribose-phosphate aldolase (n°2) |
| Dihydroxy-acid dehydratase | Dihydroxy-acid dehydratase | Dihydroxy-acid dehydratase |
| Dimodular nonribosomal peptide synthase | Dimodular nonribosomal peptide synthase | Dimodular nonribosomal peptide synthase |
| dITP/XTP pyrophosphatase | dITP/XTP pyrophosphatase | dITP/XTP pyrophosphatase |
| DNA topoisomerase 3 | DNA topoisomerase 3 | DNA topoisomerase 3 |
| DNA-binding transcriptional activator PspC | DNA-binding transcriptional activator PspC | DNA-binding transcriptional activator PspC |
| DNA-binding transcriptional regulator IlvY | DNA-binding transcriptional regulator IlvY | DNA-binding transcriptional regulator IlvY |
| dTDP-4-dehydrorhamnose 3,5-epimerase |  |  |
| dTDP-4-dehydrorhamnose reductase |  |  |
| dTDP-glucose 4,6-dehydratase |  |  |
| Eco57I restriction-modification methylase | Eco57I restriction-modification methylase | Eco57I restriction-modification methylase |
|  | Endoglucanase precursor |  |
| Exo-glucosaminidase LytG precursor | Exo-glucosaminidase LytG precursor | Exo-glucosaminidase LytG precursor |
| Fic/DOC family protein (n°2) | Fic/DOC family protein (n°2) | Fic/DOC family protein (n°2) |
|  | FMN reductase (NADPH) |  |
| Fructosamine deglycase FrlB | Fructosamine deglycase FrlB | Fructosamine deglycase FrlB |
| Fructosamine kinase FrlD | Fructosamine kinase FrlD | Fructosamine kinase FrlD |
| General stress protein A (n°2) | General stress protein A (n°2) | General stress protein A (n°2) |
| Glucose uptake protein GlcU | Glucose uptake protein GlcU | Glucose uptake protein GlcU |
| Glucose-1-phosphate thymidylyltransferase |  |  |
| Glutaminase | Glutaminase | Glutaminase |
| Glutaredoxin-like protein NrdH | Glutaredoxin-like protein NrdH | Glutaredoxin-like protein NrdH |
| Glutathione amide reductase | Glutathione amide reductase | Glutathione amide reductase |
| Glycosyl transferase family 8 (n°2) | Glycosyl transferase family 8 (n°2) | Glycosyl transferase family 8 (n°2) |
| Glycosyltransferase Gtf1 | Glycosyltransferase Gtf1 | Glycosyltransferase Gtf1 |
| Glycosyltransferase-stabilizing protein Gtf2 | Glycosyltransferase-stabilizing protein Gtf2 | Glycosyltransferase-stabilizing protein Gtf2 |
| Glyoxal reductase (n°2) | Glyoxal reductase (n°2) | Glyoxal reductase (n°2) |
| Gram positive anchor | Gram positive anchor | Gram positive anchor |
| Group II intron-encoded protein LtrA | Group II intron-encoded protein LtrA | Group II intron-encoded protein LtrA |
| Group II intron, maturase-specific domain | Group II intron, maturase-specific domain | Group II intron, maturase-specific domain |
|  | H(+)/Cl(-) exchange transporter ClcA |  |
| Helix-turn-helix domain protein (n°5) | Helix-turn-helix domain protein (n°5) | Helix-turn-helix domain protein (n°5) |
| helix-turn-helix protein (n°2) | helix-turn-helix protein (n°2) | helix-turn-helix protein (n°2) |
| Histidine kinase-, DNA gyrase B-, and HSP90-like ATPase | Histidine kinase-, DNA gyrase B-, and HSP90-like ATPase | Histidine kinase-, DNA gyrase B-, and HSP90-like ATPase |
| HNH endonuclease | HNH endonuclease | HNH endonuclease |
| Holin family protein | Holin family protein | Holin family protein |
| Homocysteine S-methyltransferase | Homocysteine S-methyltransferase | Homocysteine S-methyltransferase |
| HTH-type transcriptional activator CmpR | HTH-type transcriptional activator CmpR | HTH-type transcriptional activator CmpR |
| HTH-type transcriptional regulator ImmR (n°4) | HTH-type transcriptional regulator ImmR (n°4) | HTH-type transcriptional regulator ImmR (n°4) |
| HTH-type transcriptional regulator LacR | HTH-type transcriptional regulator LacR | HTH-type transcriptional regulator LacR |
| HTH-type transcriptional repressor CzrA | HTH-type transcriptional repressor CzrA | HTH-type transcriptional repressor CzrA |
| HxlR-like helix-turn-helix |  |  |
| Hydroxyethylthiazole kinase |  |  |
| Hydroxymethylpyrimidine/phosphomethylpyrimidine kinase |  |  |
| IclR helix-turn-helix domain protein | IclR helix-turn-helix domain protein | IclR helix-turn-helix domain protein |
| Inner membrane metabolite transport protein YgcS | Inner membrane metabolite transport protein YgcS | Inner membrane metabolite transport protein YgcS |
| Integrase core domain protein (n°8) | Integrase core domain protein (n°9) | Integrase core domain protein (n°9) |
| IS66 Orf2 like protein (n°2) | IS66 Orf2 like protein (n°2) | IS66 Orf2 like protein (n°2) |
| L-arabinose isomerase | L-arabinose isomerase | L-arabinose isomerase |
| L-lactate dehydrogenase | L-lactate dehydrogenase | L-lactate dehydrogenase |
| L-ribulose-5-phosphate 4-epimerase | L-ribulose-5-phosphate 4-epimerase | L-ribulose-5-phosphate 4-epimerase |
| Lactococcin-G-processing and transport ATP-binding protein LagD | Lactococcin-G-processing and transport ATP-binding protein LagD | Lactococcin-G-processing and transport ATP-binding protein LagD |
| Lactose permease (n°2) | Lactose permease (n°2) | Lactose permease (n°2) |
| Lanthionine synthetase C-like protein | Lanthionine synthetase C-like protein | Lanthionine synthetase C-like protein |
| Levansucrase precursor |  |  |
| LexA repressor | LexA repressor | LexA repressor |
| Linear gramicidin synthase subunit B | Linear gramicidin synthase subunit B | Linear gramicidin synthase subunit B |
| Low molecular weight protein-tyrosine-phosphatase YfkJ | Low molecular weight protein-tyrosine-phosphatase YfkJ | Low molecular weight protein-tyrosine-phosphatase YfkJ |
| LytTr DNA-binding domain protein | LytTr DNA-binding domain protein | LytTr DNA-binding domain protein |
| Macrolide export ATP-binding/permease protein MacB (n°2) | Macrolide export ATP-binding/permease protein MacB (n°2) | Macrolide export ATP-binding/permease protein MacB (n°2) |
| Major cell-surface adhesin PAc precursor | Major cell-surface adhesin PAc precursor | Major cell-surface adhesin PAc precursor |
| Major Facilitator Superfamily protein | Major Facilitator Superfamily protein | Major Facilitator Superfamily protein |
| Maltose O-acetyltransferase (n°2) | Maltose O-acetyltransferase (n°2) | Maltose O-acetyltransferase (n°2) |
| Matrixin | Matrixin | Matrixin |
| Mercuric resistance operon regulatory protein | Mercuric resistance operon regulatory protein | Mercuric resistance operon regulatory protein |
| Methylated-DNA--protein-cysteine methyltransferase | Methylated-DNA--protein-cysteine methyltransferase | Methylated-DNA--protein-cysteine methyltransferase |
| mRNA interferase EndoA | mRNA interferase EndoA | mRNA interferase EndoA |
| mRNA interferase PemK | mRNA interferase PemK | mRNA interferase PemK |
| Mrr restriction system protein (n°2) | Mrr restriction system protein (n°2) | Mrr restriction system protein (n°2) |
|  | Muramidase-2 precursor |  |
| multidrug efflux system subunit MdtA | multidrug efflux system subunit MdtA | multidrug efflux system subunit MdtA |
| N-(5-phosphoribosyl)anthranilate isomerase | N-(5-phosphoribosyl)anthranilate isomerase | N-(5-phosphoribosyl)anthranilate isomerase |
| N-acetylmuramoyl-L-alanine amidase sle1 precursor | N-acetylmuramoyl-L-alanine amidase sle1 precursor | N-acetylmuramoyl-L-alanine amidase sle1 precursor |
| NAD-dependent malic enzyme | NAD-dependent malic enzyme | NAD-dependent malic enzyme |
| NAD-dependent methanol dehydrogenase | NAD-dependent methanol dehydrogenase | NAD-dependent methanol dehydrogenase |
| NADH oxidase |  |  |
| NADPH-dependent FMN reductase | NADPH-dependent FMN reductase | NADPH-dependent FMN reductase |
| Nucleoside permease NupC | Nucleoside permease NupC | Nucleoside permease NupC |
| Pca regulon regulatory protein | Pca regulon regulatory protein | Pca regulon regulatory protein |
| PD-(D/E)XK nuclease family transposase | PD-(D/E)XK nuclease family transposase | PD-(D/E)XK nuclease family transposase |
| PemK-like protein | PemK-like protein | PemK-like protein |
| Peptidase family M23 | Peptidase family M23 | Peptidase family M23 |
| Peptidase propeptide and YPEB domain protein |  |  |
| Permease for cytosine/purines, uracil, thiamine, allantoin | Permease for cytosine/purines, uracil, thiamine, allantoin | Permease for cytosine/purines, uracil, thiamine, allantoin |
| Phage capsid family protein | Phage capsid family protein | Phage capsid family protein |
| Phage head-tail joining protein | Phage head-tail joining protein (n°2) | Phage head-tail joining protein |
| Phage integrase family protein (n°2) |  | Phage integrase family protein |
| Phage portal protein | Phage portal protein | Phage portal protein |
| Phage regulatory protein Rha (Phage_pRha) | Phage regulatory protein Rha (Phage_pRha) | Phage regulatory protein Rha (Phage_pRha) |
| Phage tail protein | Phage tail protein | Phage tail protein |
| Phage Terminase | Phage Terminase | Phage Terminase |
| Phage terminase, small subunit | Phage terminase, small subunit | Phage terminase, small subunit |
| Phage-related minor tail protein | Phage-related minor tail protein | Phage-related minor tail protein |
| phosphate-starvation-inducible protein PsiE | phosphate-starvation-inducible protein PsiE | phosphate-starvation-inducible protein PsiE |
| PIN domain protein | PIN domain protein | PIN domain protein |
| Plasmid pRiA4b ORF-3-like protein | Plasmid pRiA4b ORF-3-like protein | Plasmid pRiA4b ORF-3-like protein |
| Plasmid recombination enzyme | Plasmid recombination enzyme | Plasmid recombination enzyme |
|  | Poly-beta-1,6-N-acetyl-D-glucosamine synthase |  |
| preprotein translocase subunit SecA | preprotein translocase subunit SecA | preprotein translocase subunit SecA |
| preprotein translocase subunit SecY | preprotein translocase subunit SecY | preprotein translocase subunit SecY |
| Prophage endopeptidase tail | Prophage endopeptidase tail | Prophage endopeptidase tail |
| Protease synthase and sporulation negative regulatory protein PAI 1 | Protease synthase and sporulation negative regulatory protein PAI 1 | Protease synthase and sporulation negative regulatory protein PAI 1 |
| Pseudouridine kinase (n°2) | Pseudouridine kinase (n°2) | Pseudouridine kinase (n°2) |
| putative ABC transporter ATP-binding protein | putative ABC transporter ATP-binding protein | putative ABC transporter ATP-binding protein |
| putative ABC transporter ATP-binding protein YbhF | putative ABC transporter ATP-binding protein YbhF | putative ABC transporter ATP-binding protein YbhF |
|  | Putative arginine/ornithine antiporter |  |
|  | putative cadmium-transporting ATPase |  |
| Putative acetyltransferase |  |  |
| putative endopeptidase YafL precursor | putative endopeptidase YafL precursor | putative endopeptidase YafL precursor |
| Putative glycosyltransferase EpsH | Putative glycosyltransferase EpsH | Putative glycosyltransferase EpsH |
| putative glycosyltransferase EpsJ (n°3) | putative glycosyltransferase EpsJ (n°5) | putative glycosyltransferase EpsJ (n°5) |
| putative glycosyltransferase YkoT | putative glycosyltransferase YkoT | putative glycosyltransferase YkoT |
| putative HTH-type transcriptional regulator YurK (n°2) | putative HTH-type transcriptional regulator YurK (n°2) | putative HTH-type transcriptional regulator YurK (n°2) |
| Putative mannose-6-phosphate isomerase YvyI | Putative mannose-6-phosphate isomerase YvyI | Putative mannose-6-phosphate isomerase YvyI |
| putative metabolite transport protein CsbC | putative metabolite transport protein CsbC | putative metabolite transport protein CsbC |
| putative MFS-type transporter YhjX | putative MFS-type transporter YhjX | putative MFS-type transporter YhjX |
| putative Mg(2+) transport ATPase | putative Mg(2+) transport ATPase | putative Mg(2+) transport ATPase |
| Putative N-acetyl-LL-diaminopimelate aminotransferase (n°2) | Putative N-acetyl-LL-diaminopimelate aminotransferase (n°3) | Putative N-acetyl-LL-diaminopimelate aminotransferase (n°2) |
| putative oxalyl-CoA decarboxylase | putative oxalyl-CoA decarboxylase | putative oxalyl-CoA decarboxylase |
| putative oxidoreductase YtbE | putative oxidoreductase YtbE | putative oxidoreductase YtbE |
| Putative peptidoglycan binding domain protein | Putative peptidoglycan binding domain protein | Putative peptidoglycan binding domain protein |
| Putative prophage phiRv2 integrase (n°2) | Putative prophage phiRv2 integrase (n°2) | Putative prophage phiRv2 integrase (n°2) |
| putative sugar epimerase YhfK | putative sugar epimerase YhfK | putative sugar epimerase YhfK |
| putative sugar kinase YdjH | putative sugar kinase YdjH | putative sugar kinase YdjH |
| putative sugar transferase EpsL | putative sugar transferase EpsL | putative sugar transferase EpsL |
| putative transport protein HsrA | putative transport protein HsrA |  |
| Putative transposase DNA-binding domain protein (n°2) | Putative transposase DNA-binding domain protein (n°2) | Putative transposase DNA-binding domain protein (n°2) |
| putative type I restriction enzymeP M protein | putative type I restriction enzymeP M protein | putative type I restriction enzymeP M protein |
| Pyrimidine-specific ribonucleoside hydrolase RihA | Pyrimidine-specific ribonucleoside hydrolase RihA | Pyrimidine-specific ribonucleoside hydrolase RihA |
| Pyrroline-5-carboxylate reductase | Pyrroline-5-carboxylate reductase | Pyrroline-5-carboxylate reductase |
| Quinone oxidoreductase 2 (n°2) | Quinone oxidoreductase 2 | Quinone oxidoreductase 2 |
| Relaxase/Mobilization nuclease domain protein (n°3) | Relaxase/Mobilization nuclease domain protein (n°3) | Relaxase/Mobilization nuclease domain protein (n°3) |
| RelB antitoxin | RelB antitoxin | RelB antitoxin |
| Replication initiation and membrane attachment (n°2) | Replication initiation and membrane attachment (n°2) | Replication initiation and membrane attachment (n°2) |
| Replication initiation factor | Replication initiation factor | Replication initiation factor |
| Replication protein (n°2) | Replication protein (n°2) | Replication protein (n°2) |
| Response regulator ArlR | Response regulator ArlR | Response regulator ArlR |
| Restriction enzyme BgcI subunit alpha | Restriction enzyme BgcI subunit alpha | Restriction enzyme BgcI subunit alpha |
| Restriction enzyme BgcI subunit beta | Restriction enzyme BgcI subunit beta | Restriction enzyme BgcI subunit beta |
| Reverse transcriptase (RNA-dependent DNA polymerase) | Reverse transcriptase (RNA-dependent DNA polymerase) | Reverse transcriptase (RNA-dependent DNA polymerase) |
| Riboflavin biosynthesis protein RibBA | Riboflavin biosynthesis protein RibBA | Riboflavin biosynthesis protein RibBA |
| Riboflavin biosynthesis protein RibD | Riboflavin biosynthesis protein RibD | Riboflavin biosynthesis protein RibD |
| Riboflavin synthase | Riboflavin synthase | Riboflavin synthase |
| ribulokinase | ribulokinase | ribulokinase |
| RNA polymerase sigma factor FliA | RNA polymerase sigma factor FliA | RNA polymerase sigma factor FliA |
| rRNA adenine N-6-methyltransferase | rRNA adenine N-6-methyltransferase | rRNA adenine N-6-methyltransferase |
| Sensor histidine kinase DcuS | Sensor histidine kinase DcuS | Sensor histidine kinase DcuS |
| Sensor histidine kinase YpdA |  |  |
| Signal transduction histidine-protein kinase ArlS | Signal transduction histidine-protein kinase ArlS | Signal transduction histidine-protein kinase ArlS |
| site-specific tyrosine recombinase XerC (n°3) | site-specific tyrosine recombinase XerC (n°3) | site-specific tyrosine recombinase XerC (n°3) |
| SkfA peptide export ATP-binding protein SkfE (n°2) | SkfA peptide export ATP-binding protein SkfE | SkfA peptide export ATP-binding protein SkfE |
| Sodium Bile acid symporter family protein (n°2) | Sodium Bile acid symporter family protein (n°2) | Sodium Bile acid symporter family protein (n°2) |
| Sodium, potassium, lithium and rubidium/H(+) antiporter | Sodium, potassium, lithium and rubidium/H(+) antiporter | Sodium, potassium, lithium and rubidium/H(+) antiporter |
| Sorbitol operon regulator | Sorbitol operon regulator | Sorbitol operon regulator |
| Succinate semialdehyde dehydrogenase [NAD(P)+] Sad | Succinate semialdehyde dehydrogenase [NAD(P)+] Sad | Succinate semialdehyde dehydrogenase [NAD(P)+] Sad |
| Tetracycline resistance protein TetO | Tetracycline resistance protein TetO | Tetracycline resistance protein TetO |
| Thiamine-phosphate synthase |  | Thioredoxin |
| Thioredoxin | Thioredoxin | TM2 domain protein |
| TM2 domain protein | TM2 domain protein | Toxin A (n°4) |
| Toxin A (n°4) | Toxin A (n°4) |  |
| toxin MazF | toxin MazF | toxin MazF |
| Toxin YoeB | Toxin YoeB | Toxin YoeB |
|  | Transcriptional activatory protein AadR |  |
| transcriptional regulator BetI | transcriptional regulator BetI | transcriptional regulator BetI |
| transcriptional repressor DicA | transcriptional repressor DicA | transcriptional repressor DicA |
| Transcriptional repressor SdpR | Transcriptional repressor SdpR | Transcriptional repressor SdpR |
| Transposase (n°2) | Transposase (n°2) | Transposase (n°2) |
| Transposase DDE domain protein (n°7) | Transposase DDE domain protein (n°7) | Transposase DDE domain protein (n°7) |
| Transposase from transposon Tn916 (n°2) | Transposase from transposon Tn916 (n°2) | Transposase from transposon Tn916 (n°2) |
| Transposase IS200 like protein | Transposase IS200 like protein | Transposase IS200 like protein |
| Transposase IS66 family protein | Transposase IS66 family protein | Transposase IS66 family protein |
| transposase/IS protein (n°2) | transposase/IS protein (n°2) | transposase/IS protein (n°2) |
| Triosephosphate isomerase |  |  |
| Type I phosphodiesterase / nucleotide pyrophosphatase | Type I phosphodiesterase / nucleotide pyrophosphatase | Type I phosphodiesterase / nucleotide pyrophosphatase |
| Type I restriction modification DNA specificity domain protein (n°2) | Type I restriction modification DNA specificity domain protein (n°2) | Type I restriction modification DNA specificity domain protein (n°2) |
| Type III restriction enzyme, res subunit (n°2) | Type III restriction enzyme, res subunit (n°2) | Type III restriction enzyme, res subunit (n°2) |
| Type IV secretory system Conjugative DNA transfer | Type IV secretory system Conjugative DNA transfer | Type IV secretory system Conjugative DNA transfer |
| Type-1 restriction enzyme R protein | Type-1 restriction enzyme R protein | Type-1 restriction enzyme R protein |
| Tyrocidine synthase 3 | Tyrocidine synthase 3 | Tyrocidine synthase 3 |
| Tyrosine recombinase XerC (n°4) | Tyrosine recombinase XerC (n°4) | Tyrosine recombinase XerC (n°4) |
| UDP-D-galactose:(glucosyl)lipopolysaccharide-1, 6-D-galactosyltransferase | UDP-D-galactose:(glucosyl)lipopolysaccharide-1, 6-D-galactosyltransferase | UDP-D-galactose:(glucosyl)lipopolysaccharide-1, 6-D-galactosyltransferase |
| UDP-galactopyranose mutase | UDP-galactopyranose mutase | UDP-galactopyranose mutase |
| UDP-Glc:alpha-D-GlcNAc-diphosphoundecaprenol beta-1,3-glucosyltransferase WfgD | UDP-Glc:alpha-D-GlcNAc-diphosphoundecaprenol beta-1,3-glucosyltransferase WfgD | UDP-Glc:alpha-D-GlcNAc-diphosphoundecaprenol beta-1,3-glucosyltransferase WfgD |
| Vacuole effluxer Atg22 like protein | Vacuole effluxer Atg22 like protein | Vacuole effluxer Atg22 like protein |
| VanZ like family protein | VanZ like family protein | VanZ like family protein |
| Xylulose kinase |  |  |
| YopX protein | YopX protein | YopX protein |
| Yqey-like protein | Yqey-like protein | Yqey-like protein |

**Supplementary Table S3.** Unique individual genes in the *L. reuteri* poultry/human lineage VI belonging to the major functional classes, as illustrated in Figure 3.

| **Unique genes Human-Poultry lineage VI** | **Unique genes Human lineage VI, compared to Poultry VI** |
| --- | --- |
| Transposase IS116/IS110/IS902 family protein | [Citrate [pro-3S]-lyase] ligase |
| phosphoglycerate mutase | 2-(5-triphosphoribosyl)-3-dephosphocoenzyme- A synthase |
| Tyrosine-protein kinase YwqD | 2-(S)-hydroxypropyl-CoM dehydrogenase |
| Capsular polysaccharide type 8 biosynthesis protein cap8A | 2-C-methyl-D-erythritol 4-phosphate cytidylyltransferase |
| ASCH domain protein | 2-dehydropantoate 2-reductase (n°3) |
| Transcriptional activatory protein AadR | 23S rRNA (guanosine-2-O-)-methyltransferase RlmB |
| Copper chaperone CopZ | 3-ketoacyl-(acyl-carrier-protein) reductase |
| Zinc-transporting ATPase | 30S ribosomal protein S14 |
| Bacterial low temperature requirement A protein (LtrA) | 5TMR of 5TMR-LYT |
| ABC transporter ATP-binding protein YxdL | AAA-like domain protein |
| Bacitracin export permease protein BceB | AB hydrolase superfamily protein YdjP |
| HTH-type transcriptional regulator ImmR | ABC transporter substrate binding protein |
| Phage integrase family protein | ABC-2 family transporter protein |
| Putative HTH-type transcriptional regulator YwnA | Acetate kinase |
| Folate transporter FolT | Acetyltransferase (GNAT) family protein (n°2) |
| Quinone oxidoreductase 2 | Acyltransferase family protein |
| L-2-hydroxyisocaproate dehydrogenase | Adenosine monophosphate-protein transferase SoFic |
| 2-hydroxyhexa-2-4-dienoate hydratase | Adenosylcobalamin-dependent ribonucleoside-triphosphate reductase |
| Alpha/beta hydrolase family protein | Aldehyde-alcohol dehydrogenase (n°2) |
| Branched-chain amino acid transport protein (AzlD) | Alpha-D-kanosaminyltransferase |
| AzlC protein | Alpha-ribazole phosphatase |
| Deoxyribonucleoside regulator | Amidohydrolase family protein |
| Adenosine monophosphate-protein transferase SoFic | Amidophosphoribosyltransferase precursor |
| GTP pyrophosphokinase YwaC | Amino-acid permease RocC |
| Response regulator MprA | Ammonia channel precursor |
| Signal transduction histidine-protein kinase BaeS | anaerobic benzoate catabolism transcriptional regulator (n°3) |
| Carbamoyl-phosphate synthase large chain | Arabinose metabolism transcriptional repressor |
| Carbamoyl-phosphate synthase small chain | Arginine/agmatine antiporter (n°2) |
| Riboflavin biosynthesis protein RibBA | Arginine/ornithine antiporter |
| HTH-type transcriptional activator RhaS | aromatic amino acid aminotransferase |
| Threonine synthase | Arsenate-mycothiol transferase ArsC2 (n°2) |
| Homoserine dehydrogenase | Aryl-alcohol dehydrogenase |
| Homoserine kinase | ATP synthase subunits region ORF 6 |
| Phosphoenolpyruvate synthase | ATP-dependent Clp protease proteolytic subunit 1 (n°2) |
| Potassium lithium and rubidium/H(+) antiporter | AzlC protein |
| High-affinity gluconate transporter | Bacterial low temperature requirement A protein (LtrA) (n°2) |
| Glycerol-3-phosphate acyltransferase | Beta-1,6-galactofuranosyltransferase WbbI |
| Flavodoxin | Beta-galactosidase LacZ |
| Helix-turn-helix protein | Bifunctional adenosylcobalamin biosynthesis protein CobU |
|  | Bifunctional nicotinamide mononucleotide adenylyltransferase/ADP-ribose pyrophosphatase |
|  | Bifunctional transcriptional activator/DNA repair enzyme AdaA |
|  | C protein alpha-antigen precursor |
|  | Cadmium resistance transcriptional regulatory protein CadC |
|  | Cadmium resistance transporter (n°2) |
|  | Capsular polysaccharide type 8 biosynthesis protein cap8A |
|  | Carbohydrate diacid regulator |
|  | Carbon dioxide concentrating mechanism protein CcmL |
|  | Caudovirus prohead protease |
|  | CDP-glycerol:poly(glycerophosphate) glycerophosphotransferase |
|  | chaperone protein HscA |
|  | Choloylglycine hydrolase |
|  | Chromosome partition protein Smc |
|  | Citrate lyase acyl carrier protein |
|  | Citrate lyase alpha chain |
|  | Citrate lyase subunit beta |
|  | Cob(I)yrinic acid a,c-diamide adenosyltransferase (n°2) |
|  | cobalamin biosynthesis protein |
|  | cobalamin biosynthesis protein CbiG |
|  | Cobalamin synthase |
|  | Cobalt import ATP-binding protein CbiO |
|  | Cobalt transport protein CbiM precursor |
|  | Cobalt transport protein CbiN |
|  | Cobalt transport protein CbiQ |
|  | Cobalt-precorrin-2 C(20)-methyltransferase |
|  | Cobalt-precorrin-3B C(17)-methyltransferase |
|  | Cobalt-precorrin-4 C(11)-methyltransferase |
|  | cobalt-precorrin-6A synthase |
|  | Cobalt-precorrin-8X methylmutase |
|  | Cobyric acid synthase |
|  | Cobyrinic acid A,C-diamide synthase |
|  | Cytochrome b5-like Heme/Steroid binding domain protein |
|  | cytosine permease |
|  | D-lactate dehydrogenase |
|  | D-methionine-binding lipoprotein MetQ precursor |
|  | Delta-aminolevulinic acid dehydratase |
|  | Deoxyribose-phosphate aldolase 1 |
|  | dihydropteridine reductase |
|  | Diol dehydratase-reactivating factor alpha subunit |
|  | DNA adenine methyltransferase YhdJ |
|  | DNA-3-methyladenine glycosylase 1 |
|  | DNA-binding transcriptional activator PspC |
|  | DNA-invertase hin |
|  | Double zinc ribbon |
|  | dTDP-4-dehydrorhamnose 3,5-epimerase |
|  | dTDP-4-dehydrorhamnose reductase (n°3) |
|  | dTDP-glucose 4,6-dehydratase (n°2) |
|  | EamA-like transporter family protein (n°3) |
|  | EcoKI restriction-modification system protein HsdS (n°2) |
|  | Endoglucanase precursor |
|  | ERF superfamily protein |
|  | Excisionase from transposon Tn916 |
|  | Exo-glucosaminidase LytG precursor |
|  | Ferrous iron transport protein B |
|  | Flavodoxin |
|  | FMN reductase (NADPH) |
|  | FMN-dependent NADPH-azoreductase |
|  | Galactofuranosyl transferase GlfT1 |
|  | Glucose-1-phosphate thymidylyltransferase |
|  | Glucosyltransferase-SI precursor |
|  | Glutamate-1-semialdehyde 2,1-aminomutase |
|  | Glutaminase |
|  | Glutamyl-tRNA reductase |
|  | Glutathione amide reductase |
|  | Glycosyl hydrolases family 25 (n°2) |
|  | Glyoxal reductase |
|  | Helix-turn-helix domain protein (n°2) |
|  | helix-turn-helix protein (n°2) |
|  | HNH endonuclease (n°2) |
|  | Holin family protein (n°3) |
|  | Homocysteine S-methyltransferase |
|  | HTH-type transcriptional activator CmpR |
|  | HTH-type transcriptional activator TipA |
|  | HTH-type transcriptional regulator ImmR |
|  | HTH-type transcriptional regulator LacR |
|  | HTH-type transcriptional regulator SinR |
|  | HTH-type transcriptional regulator Xre |
|  | HTH-type transcriptional repressor CzrA |
|  | Inner membrane metabolite transport protein YgcS |
|  | Integrase core domain protein (n°8) |
|  | IS66 Orf2 like protein |
|  | L-arabinose isomerase |
|  | L-lactate dehydrogenase |
|  | L-ribulose-5-phosphate 4-epimerase |
|  | Lactose permease |
|  | Low molecular weight protein-tyrosine-phosphatase YfkJ |
|  | Major carboxysome shell protein 1C |
|  | Maltose O-acetyltransferase |
|  | Maltose O-acetyltransferase |
|  | MarR family protein |
|  | Matrixin |
|  | Methylated-DNA--protein-cysteine methyltransferase |
|  | Modification methylase DpnIIB |
|  | Molybdenum cofactor biosynthesis protein B |
|  | Molybdopterin molybdenumtransferase |
|  | Molybdopterin-guanine dinucleotide biosynthesis adapter protein |
|  | molybdopterin-guanine dinucleotide biosynthesis protein MobA |
|  | MucBP domain protein |
|  | Multidrug resistance protein 3 |
|  | Muramidase-2 precursor |
|  | N-6 DNA Methylase |
|  | NAD-dependent malic enzyme |
|  | NADH oxidase |
|  | Nicotinate phosphoribosyltransferase pncB2 |
|  | Nicotinate-nucleotide--dimethylbenzimidazole phosphoribosyltransferase |
|  | Nitrate reductase delta subunit |
|  | Nitrate reductase-like protein NarX |
|  | Pca regulon regulatory protein |
|  | PD-(D/E)XK nuclease superfamily protein (n°2) |
|  | Peptidase family M23 |
|  | Peptidase family M23 |
|  | Peptidase propeptide and YPEB domain protein |
|  | Peptidoglycan endopeptidase RipB precursor |
|  | PglZ domain protein |
|  | Phage antirepressor protein KilAC domain protein |
|  | Phage capsid family protein (n°4) |
|  | Phage gp6-like head-tail connector protein (n°4) |
|  | Phage head-tail joining protein (n°5) |
|  | Phage portal protein (n°3) |
|  | Phage tail protein |
|  | Phage Terminase (n°3) |
|  | Phage terminase, small subunit (n°3) |
|  | Phage-related minor tail protein |
|  | Phosphate propanoyltransferase |
|  | phosphate-starvation-inducible protein PsiE |
|  | Phosphoribosylformylglycinamidine synthase 2 |
|  | Phosphoserine phosphatase 1 |
|  | Poly-beta-1,6-N-acetyl-D-glucosamine synthase |
|  | Porphobilinogen deaminase |
|  | Precorrin-2 dehydrogenase |
|  | Precorrin-6A reductase |
|  | Primosomal protein DnaI |
|  | Propanediol dehydratase large subunit |
|  | Propanediol dehydratase medium subunit |
|  | Propanediol dehydratase small subunit |
|  | Propanediol utilization protein PduA |
|  | Propanediol utilization protein PduB |
|  | Propanediol utilization protein PduU |
|  | Propanediol utilization protein PduV |
|  | Prophage endopeptidase tail |
|  | putative ABC transporter ATP-binding protein (n°3) |
|  | putative ABC transporter ATP-binding protein YbhF |
|  | Putative acetyltransferase |
|  | putative amino acid permease YhdG |
|  | putative amino-acid racemase |
|  | putative cobalt-precorrin-6Y C(15)-methyltransferase [decarboxylating] |
|  | putative cobalt-precorrin-6Y C(5)-methyltransferase |
|  | Putative glycosyltransferase EpsH |
|  | putative glycosyltransferase EpsJ (n°2) |
|  | putative HTH-type transcriptional regulator YjiR |
|  | putative mannose-6-phosphate isomerase GmuF |
|  | Putative membrane protein insertion efficiency factor |
|  | putative metabolite transport protein CsbC |
|  | putative MFS-type transporter YhjX |
|  | putative Mg(2+) transport ATPase |
|  | putative multidrug resistance protein EmrY |
|  | putative N-acetyltransferase YvbK |
|  | putative oxalyl-CoA decarboxylase |
|  | putative oxidoreductase YtbE |
|  | putative oxidoreductase/MSMEI_2347 |
|  | putative poly(glycerol-phosphate) alpha-glucosyltransferase |
|  | Putative teichuronic acid biosynthesis glycosyltransferase TuaG |
|  | putative thiol peroxidase |
|  | putative transport protein HsrA |
|  | putative transposase |
|  | Putative transposase DNA-binding domain protein |
|  | putative two-component response-regulatory protein YehT |
|  | Putative undecaprenyl-phosphate N-acetylgalactosaminyl 1-phosphate transferase |
|  | Quinone oxidoreductase 2 |
|  | Recombinase |
|  | Relaxase/Mobilization nuclease domain protein |
|  | Respiratory nitrate reductase 1 alpha chain |
|  | Respiratory nitrate reductase 1 beta chain |
|  | Response regulator ArlR |
|  | ribulokinase |
|  | RNA helicase |
|  | RNA polymerase factor sigma-70 |
|  | RNA polymerase sigma factor SigF |
|  | Serine/threonine exchanger SteT |
|  | Signal transduction histidine-protein kinase ArlS |
|  | Single-stranded DNA-binding protein ssb |
|  | Sirohydrochlorin cobaltochelatase |
|  | site-specific tyrosine recombinase XerC (n°2) |
|  | ski2-like helicase (n°2) |
|  | Sodium Bile acid symporter family protein |
|  | Sorbitol operon regulator |
|  | Teichuronic acid biosynthesis protein TuaB |
|  | Threonine-phosphate decarboxylase |
|  | Toxin Doc |
|  | transcriptional regulator SlyA |
|  | transposase (n°4) |
|  | Transposase DDE domain protein (n°4) |
|  | Transposase from transposon Tn916 |
|  | Transposase IS200 like protein (n°2) |
|  | Transposase IS66 family protein (n°3) |
|  | transposase/IS protein |
|  | Transposon gamma-delta resolvase (n°3) |
|  | Transposon Tn3 resolvase (n°2) |
|  | Type I phosphodiesterase / nucleotide pyrophosphatase |
|  | Type I restriction enzyme EcoKI M protein |
|  | Type I restriction enzyme EcoR124II R protein |
|  | Type III restriction enzyme, res subunit |
|  | Tyrosine recombinase XerC (n°2) |
|  | Tyrosine recombinase XerD |
|  | Tyrosine-protein phosphatase precursor |
|  | UDP-Glc:alpha-D-GlcNAc-diphosphoundecaprenol beta-1,3-glucosyltransferase WfgD |
|  | UDP-glucose 4-epimerase |
|  | Undecaprenyl-phosphate 4-deoxy-4-formamido-L-arabinose transferase |
|  | Uracil DNA glycosylase superfamily protein |
|  | Uroporphyrinogen-III C-methyltransferase |
|  | uroporphyrinogen-III synthase |
|  | VanZ like family protein (n°2) |
|  | Vibriobactin-specific isochorismatase |
|  | VRR-NUC domain protein (n°2) |
|  | Xylulose kinase |
|  | YSIRK type signal peptide |

**Supplementary Table S4.** AMR genes detected in the genomes of *L. reuteri* strains isolated from different hosts and published in NCBI.

| **Strain** | **Origin** | **ERM** | **PEN** | **TET** | **VAN** | **CIP** |
| --- | --- | --- | --- | --- | --- | --- |
| P43 | Chicken |  | *ponA, pbpX, pbpF, pbpB* | *tetA, tetO* |  | *gyrA, gyrB, parB, parC, parE, prmA, prmC* |
| An71 | Chicken |  | *ponA, pbpX, pbpF, pbpB* | *tetA, tetO, tetW* |  | *gyrA, gyrB, parB, parC, parE, prmA, prmC* |
| An166 | Chicken |  | *ponA, pbpX, pbpF, pbpB* | *tetA, tetO, tetW* |  | *gyrA, gyrB, parB, parC, parE, prmA, prmC* |
| 1366 | Chicken |  | *ponA, pbpX, pbpF, pbpB* | *tetA, tetO* |  | *gyrA, gyrB, parB, parC, parE, prmA, prmC* |
| JCM1081 | Chicken |  | *ponA, pbpX, pbpF, pbpB* | *tetA, tetO* |  | *gyrA, gyrB, parB, parC, parE, prmA, prmC* |
| CSF8 | Chicken | *erm*(B) | *ponA, pbpX, pbpF, pbpB* | *tetA, tetO* |  | *gyrA, gyrB, parB, parC, parE, prmA, prmC* |
| IRT | Human |  | *ponA, pbpX, pbpF, pbpB* | *tetA, tetO* |  | *gyrA, gyrB, parB, parC, parE, prmA, prmC* |
| JCM 1112 | Human |  | *ponA, pbpX, pbpF, pbpB* | *tetA, tetO* |  | *gyrA, gyrB, parB, parC, parE, prmA, prmC* |
| CF48_3A | Human |  | *ponA, pbpX, pbpF, pbpB* | *tetA, tetO, tetW* |  | *gyrA, gyrB, parB, parC, parE, prmA, prmC* |
| MM2_3 | Human |  | *ponA, pbpX, pbpF, pbpB* | *tetA, tetO, tetC* |  | *gyrA, gyrB, parB, parC, parE, prmA, prmC* |
| 100_23 | Rat |  | *ponA, pbpX, pbpF, pbpB* | *tetA, tetO* |  | *gyrA, gyrB, parB, parC, parE, prmA, prmC* |
| I49 | Mouse |  | *ponA, pbpX, pbpF, pbpB* | *tetA, tetO* |  | *gyrA, gyrB, parB, parC, parE, prmA, prmC* |
| mlc3 | Mouse |  | *ponA, pbpX, pbpF, pbpB* | *tetA, tetO* |  | *gyrA, gyrB, parB, parC, parE, prmA, prmC* |
| lpuph | Mouse |  | *ponA, pbpX, pbpF, pbpB* | *tetA, tetO* |  | *gyrA, gyrB, parB, parC, parE, prmA, prmC* |
| ATCC53608 | Pig |  | *ponA, pbpX, pbpF, pbpB* | *tetA, tetO* | *vanH* | *gyrA, gyrB, parB, parC, parE, prmA, prmC* |
| I5007 | Pig |  | *ponA, pbpX, pbpF, pbpB* | *tetA, tetO, tetM, tetW* |  | *gyrA, gyrB, parB, parC, parE, prmA, prmC* |
| ZLR003 | Pig |  | *ponA, pbpX, pbpF, pbpB* | *tetA, tetO, tetW, tetL* |  | *gyrA, gyrB, parB, parC, parE, prmA, prmC* |
| 20_02 | Pig |  | *ponA, pbpX, pbpF, pbpB* | *tetA, tetO, tetM, tetW* |  | *gyrA, gyrB, parB, parC, parE, prmA, prmC* |
| CRL1098 | Sourdough |  | *ponA, pbpX, pbpF, pbpB* | *tetA, tetO* |  | *gyrA, gyrB, parB, parC, parE, prmA, prmC* |
| TMW1.656 | Sourdough |  | *pbpX, pbpF, pbpB, pbpA, pbpG* | *tetA, tetO* |  | *gyrA, gyrB, parB, parC, parE, prmA, prmC* |
| TMW1.112 | Sourdough |  | *pbpX, pbpF, pbpB, pbpA* | *tetA, tetO* |  | *gyrA, gyrB, parB, parC, parE, prmA, prmC* |
| LTH5448 | Sourdough |  | *ponA, pbpX, pbpF, pbpB* | *tetA, tetO* |  | *parB, parC, parE, prmA, prmC* |
| LR1 | Goat |  | *ponA, pbpX, pbpF, pbpB* | *tetA, tetO* |  | *gyrA, gyrB, parB, parC, parE, prmA, prmC* |
| LR2 | Goat |  | *ponA, pbpX, pbpF, pbpB* | *tetA, tetO* |  | *gyrA, gyrB, parB, parC, parE, prmA, prmC* |
| LR3 | Goat |  | *ponA, pbpX, pbpF, pbpB* | *tetA, tetO* |  | *gyrA, gyrB, parB, parC, parE, prmA, prmC* |
| LR11 | Goat |  | *ponA, pbpX, pbpF, pbpB* | *tetA, tetO* |  | *gyrA, gyrB, parB, parC, parE, prmA, prmC* |
| LR14 | Goat |  | *ponA, pbpX, pbpF, pbpB* | *tetA, tetO* |  | *gyrA, gyrB, parB, parC, parE, prmA, prmC* |
| LR6 | Sheep |  | *ponA, pbpX, pbpF, pbpB* | *tetA, tetO* |  | *gyrA, gyrB, parB, parC, parE, prmA, prmC* |
| LR7 | Sheep |  | *ponA, pbpX, pbpF, pbpB* | *tetA, tetO* |  | *gyrA, gyrB, parB, parC, parE, prmA, prmC* |
| LR8 | Sheep |  | *ponA, pbpX, pbpF, pbpB* | *tetA, tetO, tetM* |  | *gyrA, gyrB, parB, parC, parE, prmA, prmC* |
| LR9 | Sheep |  | *ponA, pbpX, pbpF, pbpB* | *tetA, tetO, tetM* |  | *gyrA, gyrB, parB, parC, parE, prmA, prmC* |
| LR4 | Cow |  | *ponA, pbpX, pbpF, pbpB* | *tetA, tetO* |  | *gyrA, gyrB, parB, parC, parE, prmA, prmC* |
| LR10 | Cow |  | *ponA, pbpX, pbpF, pbpB* | *tetA, tetO* |  | *gyrA, gyrB, parB, parC, parE, prmA, prmC* |
| LR12 | Cow |  | *ponA, pbpX, pbpF, pbpB* | *tetA, tetO* |  | *gyrA, gyrB, parB, parC, parE, prmA, prmC* |
| LR13 | Cow |  | *ponA, pbpX, pbpF, pbpB* | *tetA, tetO* |  | *gyrA, gyrB, parB, parC, parE, prmA, prmC* |
| LR17 | Horse |  | *ponA, pbpX, pbpF, pbpB* | *tetA, tetO* |  | *gyrA, gyrB, parB, parC, parE, prmA, prmC* |
| LR18 | Horse |  | *ponA, pbpX, pbpF, pbpB* | *tetA, tetO* |  | *gyrA, gyrB, parB, parC, parE, prmA, prmC* |
| LR19 | Horse |  | *ponA, pbpX, pbpF, pbpB* | *tetA, tetO, tetM* |  | *gyrA, gyrB, parB, parC, parE, prmA, prmC* |

CFX, cefotaxime; ERM, erythromycin; PEN, penicillin; TET, tetracycline; VAN; vancomycin; CIP, ciprofloxacin.

**Supplementary Table S5.** Deduced plasmid position of *ermB* and *tetW* genes associated with resistant phenotypes as results of alignment with a custom base *L. reuteri* plasmid database (Table 2).

| **Strain** | **AMR gene** | **Location** | **Plasmid** | **Query Cover** | **E value** | **% Identity** | **Description, best match** |
| --- | --- | --- | --- | --- | --- | --- | --- |
| PTA5_11 | *ermB* | NODE_74 | yes | 100% | 0 | 99.60% | NC_0035128.1 / *L.reuteri* plasmid pTE44, complete sequence |
|  | *tetW* | NODE_5 | yes | 100% | 0 | 99.95% | NC_010603.1 / *L. reuter*i ATCC55730 plasmid pLR581, complete sequence |
| PTA8_1 | *ermB* | NODE_73 | yes | 100% | 0 | 99.60% | NC_0035128.1 / *L.reuteri* plasmid pTE44, complete sequence |
|  | *tetW* | NODE_5 | yes | 100% | 0 | 99.95% | NC_010603.1/  *L. reuter*i ATCC55730 plasmid pLR581, complete sequence |
| PTA5_F4 | *tetW* | NODE_47 | yes | 100% | 0 | 99.90% | NC_010603.1 / *L. reuteri* ATCC55730 plasmid pLR581, complete sequence |
| PTA6_F1 | *ermB* | NODE_50 | yes | 100% | 0 | 99.86% | NC_0035128.1 / *L.reuteri* plasmid pTE44, complete sequence |
| PTA5_F1 | *ermB* | NODE_48 | yes | 100% | 0 | 100% | NC_010621.1 / *L. reuteri* ATCC55730 plasmid pLR585, complete sequence |
