# Supplementary material for: Isolation and Comparative Genomic Analysis of Reuterin-Producing Lactobacillus reuteri From the Chicken Gastrointestinal Tract
Source: Front Microbiol. 2020 Jun 4;11:1166. doi: 10.3389/fmicb.2020.01166 (PMC7326114; doi:10.3389/fmicb.2020.01166)
Supplement: Supplementary file 2 [file Data_Sheet_2.docx]

**Supplementary Figure S1**. ERIC PCR profiles of selected *L. reuteri* isolated in this study from chicken faeces and caecum. The strains were named PTA X, with X indicating the chicken ID, with no number being assigned to the abattoir sample; F and C indicate isolating from faeces and caecum, respectively, followed by the number of the isolated strains. Reuterin positive (+) and negative (-) strains are indicated in parenthesis. In bold, 25 trains selected for whole genome sequencing and further analysis.

**
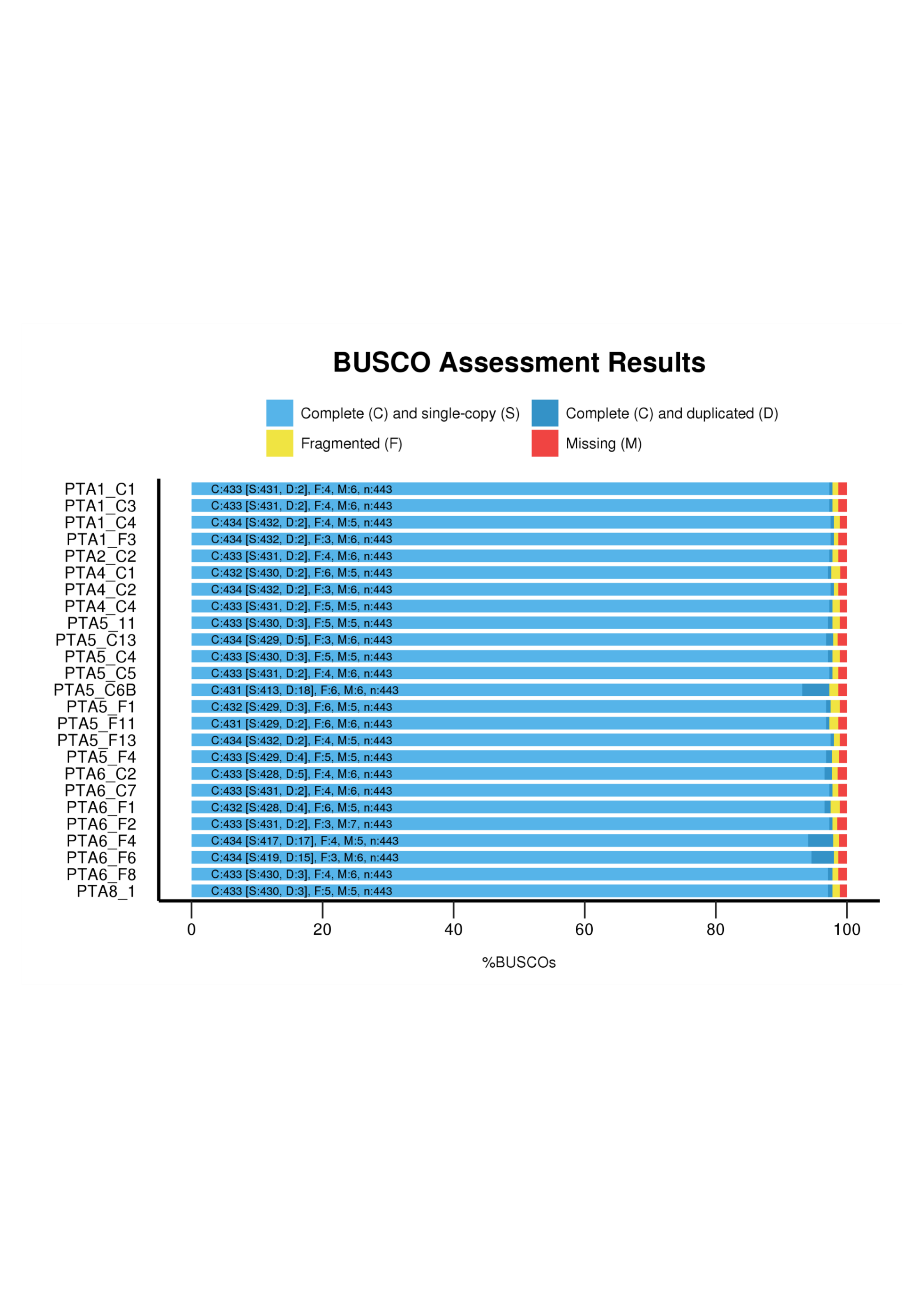
**

**Supplementary Figure S2.** BUSCO genome assembly assessment of 25 draft genomes of *L. reuteri* chicken isolates from this study.

**
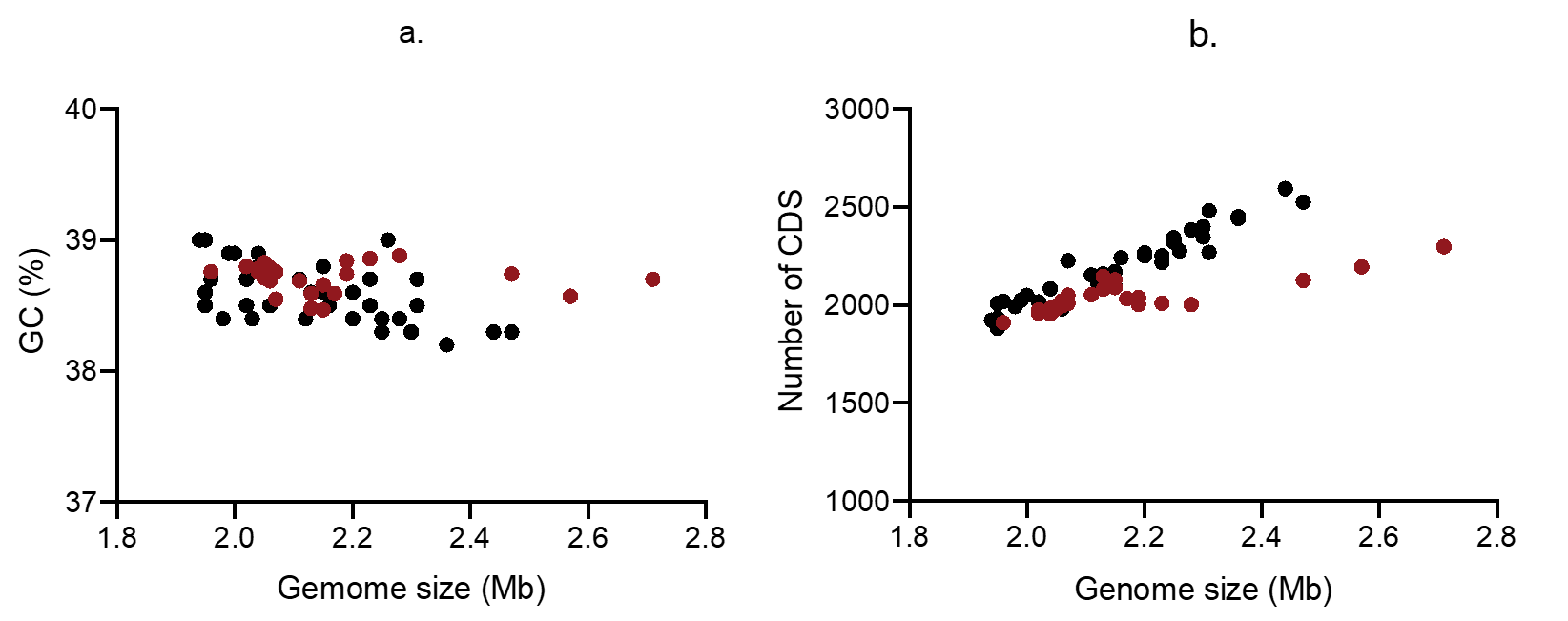
**

**Supplementary Figure S3.** Genomic characteristics of *L. reuteri* chicken isolates of this study (red dots) compared to 40 NCBI *L. reuteri* deposited genomes (black dots); a) correlation between GC content (%) and genome size; b) correlation between genome size and the number of coding sequences (CDSs).


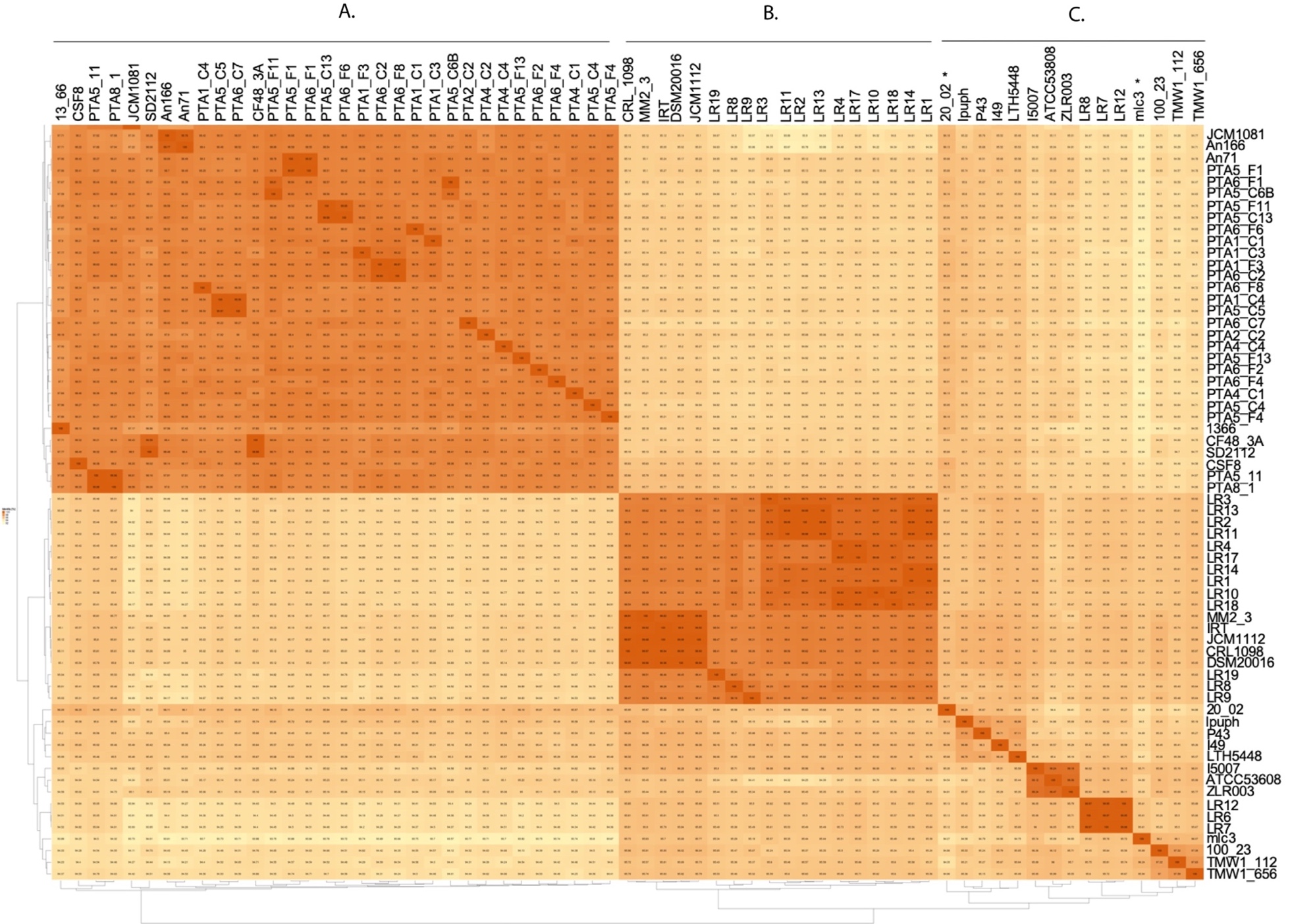


**Supplementary Figure S4.** Average nucleotide identity (ANI) of *L. reuteri* isolates calculated with EDGAR 2.3. Asterisk (*) indicate two strains belonging to cluster II in the genetic tree.

**
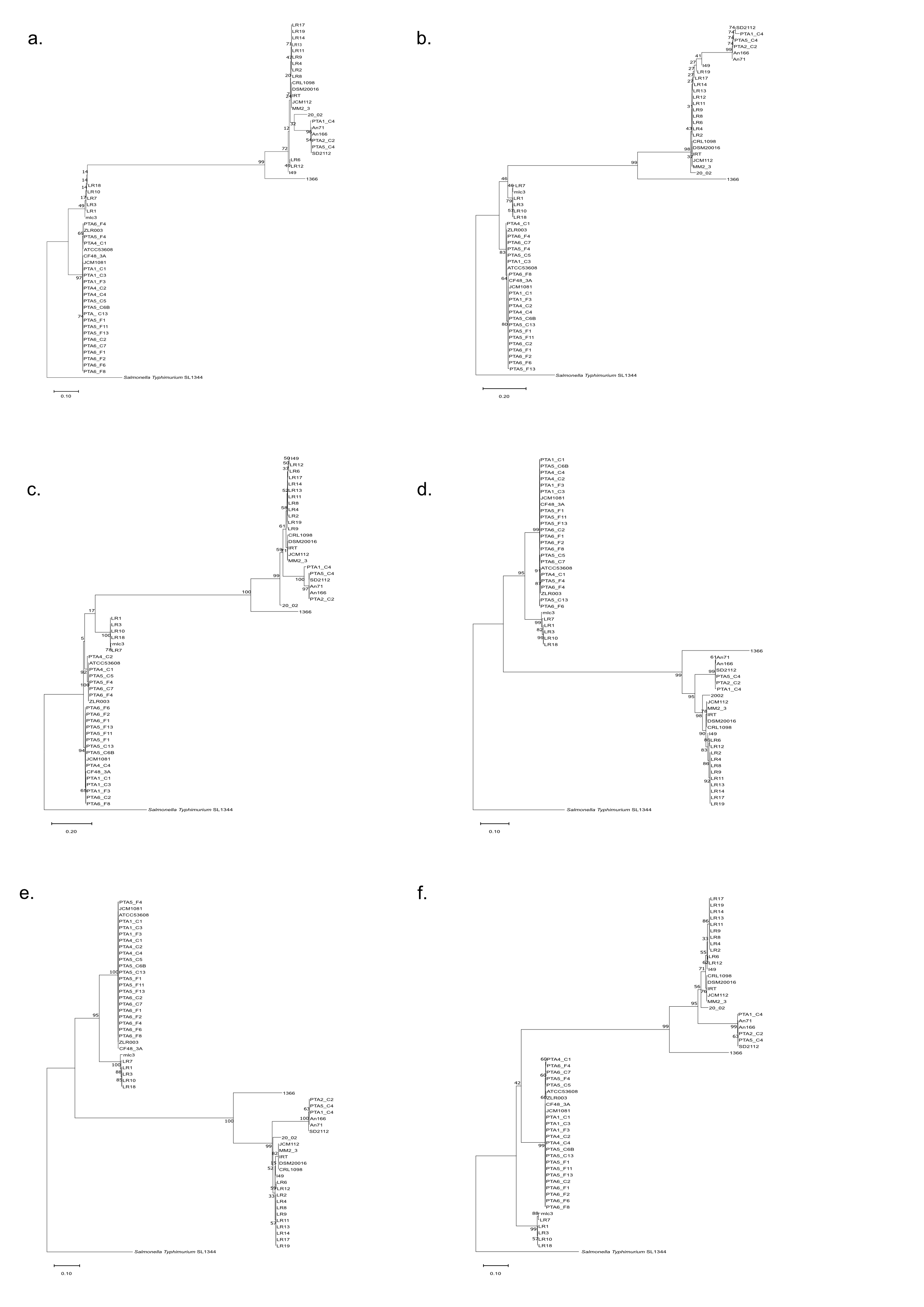
**

**Supplementary Figure S5.** Neighbor-joining tree of *pdu* genes sequences extracted from the genomes of the 55 reuterin-positive *L. reuteri* genomes analysed in this study. a.) pduA_1, b.) pduA_2, c.) pduB, d.) pduC, e.) pduD, f.) pduE.
